# Combined *In-silico* and Machine Learning Approaches Toward Predicting Arrhythmic Risk in Post-infarction Patients

**DOI:** 10.3389/fphys.2021.745349

**Published:** 2021-11-08

**Authors:** Mary M. Maleckar, Lena Myklebust, Julie Uv, Per Magne Florvaag, Vilde Strøm, Charlotte Glinge, Reza Jabbari, Niels Vejlstrup, Thomas Engstrøm, Kiril Ahtarovski, Thomas Jespersen, Jacob Tfelt-Hansen, Valeriya Naumova, Hermenegild Arevalo

**Affiliations:** ^1^Computational Physiology, Simula Research Laboratory, Oslo, Norway; ^2^Department of Cardiology, Rigshospitalet, Copenhagen University Hospital, Copenhagen, Denmark; ^3^Department of Biomedical Sciences, University of Copenhagen, Copenhagen, Denmark; ^4^Department of Forensic Medicine, Faculty of Medical Sciences, University of Copenhagen, Copenhagen, Denmark

**Keywords:** patient-specific modeling, computational cardiology, machine learning in cardiology, modeling and simulation, biophysical modeling, data augmentation, electrophysiological modeling

## Abstract

**Background:** Remodeling due to myocardial infarction (MI) significantly increases patient arrhythmic risk. Simulations using patient-specific models have shown promise in predicting personalized risk for arrhythmia. However, these are computationally- and time- intensive, hindering translation to clinical practice. Classical machine learning (ML) algorithms (such as K-nearest neighbors, Gaussian support vector machines, and decision trees) as well as neural network techniques, shown to increase prediction accuracy, can be used to predict occurrence of arrhythmia as predicted by simulations based solely on infarct and ventricular geometry. We present an initial combined image-based patient-specific *in silico* and machine learning methodology to assess risk for dangerous arrhythmia in post-infarct patients. Furthermore, we aim to demonstrate that simulation-supported data augmentation improves prediction models, combining patient data, computational simulation, and advanced statistical modeling, improving overall accuracy for arrhythmia risk assessment.

**Methods:** MRI-based computational models were constructed from 30 patients 5 days post-MI (the “baseline” population). In order to assess the utility biophysical model-supported data augmentation for improving arrhythmia prediction, we augmented the virtual baseline patient population. Each patient ventricular and ischemic geometry in the baseline population was used to create a subfamily of geometric models, resulting in an expanded set of patient models (the “augmented” population). Arrhythmia induction was attempted via programmed stimulation at 17 sites for each virtual patient corresponding to AHA LV segments and simulation outcome, “arrhythmia,” or “no-arrhythmia,” were used as ground truth for subsequent statistical prediction (machine learning, ML) models. For each patient geometric model, we measured and used choice data features: the myocardial volume and ischemic volume, as well as the segment-specific myocardial volume and ischemia percentage, as input to ML algorithms. For classical ML techniques (ML), we trained k-nearest neighbors, support vector machine, logistic regression, xgboost, and decision tree models to predict the simulation outcome from these geometric features alone. To explore neural network ML techniques, we trained both a three - and a four-hidden layer multilayer perceptron feed forward neural networks (NN), again predicting simulation outcomes from these geometric features alone. ML and NN models were trained on 70% of randomly selected segments and the remaining 30% was used for validation for both baseline and augmented populations.

**Results:** Stimulation in the baseline population (30 patient models) resulted in reentry in 21.8% of sites tested; in the augmented population (129 total patient models) reentry occurred in 13.0% of sites tested. ML and NN models ranged in mean accuracy from 0.83 to 0.86 for the baseline population, improving to 0.88 to 0.89 in all cases.

**Conclusion:** Machine learning techniques, combined with patient-specific, image-based computational simulations, can provide key clinical insights with high accuracy rapidly and efficiently. In the case of sparse or missing patient data, simulation-supported data augmentation can be employed to further improve predictive results for patient benefit. This work paves the way for using data-driven simulations for prediction of dangerous arrhythmia in MI patients.

## 1. Introduction

Ventricular arrhythmia, resulting from abnormal impulse propagation in the heart, is a leading cause of death in the industrialized world (Zipes and Wellens, 1998). Ventricular tachycardia (VT), a life-threatening regular and repetitive fast heart rhythm, frequently occurs in the setting of myocardial infarction (MI), as does the even more dangerous and disorganized ventricular fibrillation (VF), occurring when blockage in the coronary arteries impedes perfusion to the heart muscle, causing both acute and chronic damage. Implantation of a cardioverter-defibrillator (ICD) is the most effective measure for preventing lethal arrhythmias post-MI; however, ICD therapy is costly and can be associated with procedural complications, infections, device malfunctions and diminished quality of life (Zipes et al., 2016). In addition to the risks associated with ICD implantation itself, current guidelines for which patients may benefit from this intervention critically need improvement. Currently clinical criteria for identifying ICD candidates for the primary prevention of sudden cardiac death (SCD) rely almost exclusively on a nonspecific reduction in global left ventricular function (ejection fraction <35%). Only 5% of patients who meet this criterion and thus undergo device implantation receive life-saving appropriate defibrillation shocks (Smer et al., 2017). Patient-specific models can be successfully employed to improve arrhythmia risk assessment for post-MI patients. Specifically, previous work in computational cardiology has helped both in outlining the role of MI mechanistically driving arrhythmia risk, and in assessing individualized patient risk for dangerous arrhythmia (Arevalo et al., 2013, 2016). Specifically, clinical magnetic resonance imaging (MRI) with late gadolinium enhancement (LGE) can be used to construct a 3D computer model of an individual patient's heart, incorporating the patient's ventricular geometry, structural remodeling, as well as electrical properties (subcellular to organ). This patient heart, used in a series of virtual electrophysiology lab induction protocols, can be used to assess individual risk for dangerous arrhythmia post-MI and links abnormal myocardial structure to arrhythmogenicity. The above approach, and analogous methodologies for other disease states, have been making inroads with great success so far, but simulations in computational cardiology are resource- and time-intensive. Despite notable successes, in many cases their costliness hinders their translation to clinical practice for improved patient risk assessment and treatment planning.

Artificial intelligence (AI, encompassing both traditional, feature-based machine learning, as well as “deep” neural networks), has emerged as remarkably successful in tackling a wide variety of challenges in healthcare over the last decade, including in cardiology (Topol, 2019; Lopez-Jimenez et al., 2020; Erickson, 2021). In contrast to biophysical models, which can offer detailed personalized insight as outlined above but can be cumbersome with respect to computational resources, once trained, AI models can be remarkably efficient and quick to run, as well as accurate. Thus, AI is attractive for clinical timescales wherein decisions need to be made quickly on readily available computer systems. AI algorithms can learn outcomes (e.g., classify disease or assess risk) as based on key patient biomarkers (i.e., hand-engineered features), in the case of traditional machine learning, or even in the case wherein distinguishing biomarkers are unknown (in the case of deep learning/neural networks, which often provide superior accuracy and recall).

Indeed, machine learning has been used extensively in cardiovascular medicine, not least in the automatic interpretation and classification of ECG signals (Kusunose et al., 2020; Chang et al., 2021; Hicks et al., 2021; Thambawita et al., 2021; Van De Leur et al., 2021; Zhou et al., 2021). Many studies have also successfully employed ML in arrhythmia risk stratification, including advanced ML-enabled image analysis (Feeny et al., 2020; Krittanawong et al., 2020; Trayanova, 2021). Recently, ML models have been combined with biophysical modeling to assess risk for dangerous arrhythmia as well as to uncover mechanisms of rhythm disturbances and to manage treatment for affected patients (Prakosa et al., 2013; Bernard et al., 2018; Lozoya et al., 2019; Shade et al., 2020; Banus et al., 2021; Monaci et al., 2021; Sermesant et al., 2021; Trayanova, 2021). Biophysical cardiac computational modeling and ML have also increasingly been combined to focus on drug-induced proarrhythmic risk assessment, as in e.g., Yang et al. (2020) and Sahli-Costabal et al. (2020). Thus, biophysically-detailed, patient-specific models, which may offer mechanistic insight, can be combined with AI models, which offer superior speed and accuracy for predictive tasks. However, AI models often require sufficient data for optimal performance. Usage of clinical data already implies particular challenges, including practicalities of access for engineering development and necessary requirements for data protection, e.g., anonymization. However, data from clinical studies, while sufficient for traditional statistical analysis, may also simply not represent the quantity of data necessary to achieve a superior result with some AI approaches, e.g., neural networks. Furthermore, data features which are hand-selected or engineered as based on traditional clinical biomarkers may not provide optimal predictive performance.

Generally, data augmentation is a technique used to create novel examples of data by slightly altering existing data and/or creating *de novo* synthetic data from existing data for training of machine learning models. This additional data acts as a regularizer, and helps to reduce overfitting when training models, particularly neural networks. Data augmentation has been used in diverse biomedical contexts to improve model performance, see e.g., ECG classification models including GAN-enabled augmentation of ECG datasets (Golany et al., 2020; Shaker et al., 2020; Thambawita et al., 2021). Biophysical simulation-based data creation (augmentation) goes a step further, to use detailed mechanistic models, often incorporating patient-specific aspects, to increase and enrich the amount of data available to train AI models. These broadly range from e.g., biophysics-based domain adaptation methods to improve AI-enabled image processing in the brain (Gholami et al., 2018) to studies applicable to arrhythmia assessment and treatment planning in patients (Lozoya et al., 2019; Shade et al., 2020).

Computational cardiac simulations can create expanded patient data — as based on first-principles biophysics — for a single patient, or a population of patients: i.e., voltage-mapping to assess inducibility of VT post-MI, when only image-based geometries (LGE-MRI) are available. The expanded, augmented population from biophysically-detailed computational cardiology simulations can then be used to train AI models for a downstream task (in this case, assessing patient risk for dangerous arrhythmia post-MI by a series of classification models).

In this study, we present an initial combined image-based patient-specific *in silico* and machine learning methodology to assess risk for dangerous arrhythmia in post-infarct patients. Furthermore, we aim to demonstrate that simulation-supported data augmentation improves prediction models, combining patient data, computational simulation, and advanced statistical modeling, improving overall accuracy for arrhythmia risk assessment. We present a semi-automated image-based patient-specific modeling and simulation pipeline and well as data-augmentation and machine learning techniques, and show that a combined approach can provide key clinical insights with high accuracy rapidly and efficiently. In the case of sparse or missing patient data, simulation-supported data augmentation can be employed to further improve predictive results for patient benefit. This work paves the way for using data-driven simulations for prediction of dangerous arrhythmia in MI patients.

## 2. Methods and Materials

### 2.1. Image-Based Modeling Pipeline

**Figure 1:**
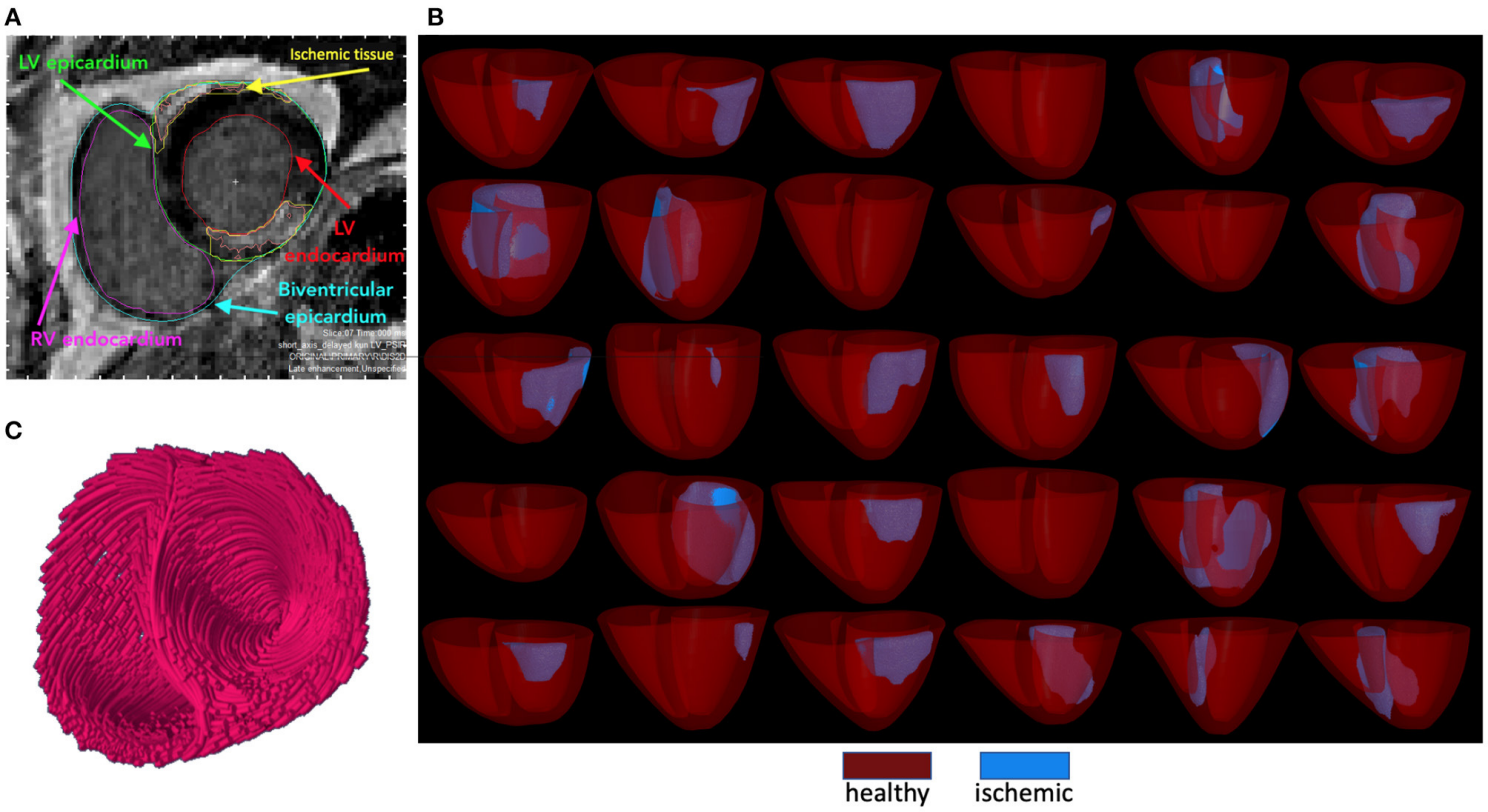
**(A)** Patient MRI and segmentation of endocardial, epicardial, and ischemic surfaces. **(B)** Rule-based fiber orientation. **(C)** The generated 30 baseline geometries with ventricles rendered semi-transparently.

Several prior studies have developed pipelines generating personalized heart models, (e.g., Vadakkumpadan et al., 2010); however, these processes have generally been time-consuming and manual. We developed and implemented a semi-automatic pipeline for generating patient-specific ventricular models (Figure 1). All steps are fully automated, with the exception of MRI segmentation, which required manual intervention. The entire pipeline is open-source and available to the public. This semi-automated pipeline involves segmentation from MRI medical images of the heart, finite element model generation, virtual myocardial fiber generation, and node reordering as preparation for continuum model electrophysiological simulations.

#### 2.1.1. Baseline Clinical Information for Initial Patient Groups

In collaboration with Rigshospitalet in Copenhagen, DK, we received access to MRI of 48 patients suffering from first-time MI (Jabbari et al., 2015; Ravn Jacobsen et al., 2020). After immediate primary percutaneous coronary intervention (PPCI), all patients underwent MRI scans 5 days post procedure. The data set available for this study was reduced to 30 patients after data assessment for quality and suitability for the image-based modeling pipeline described below.

#### 2.1.2. MRI Segmentation

Segmentation was attained using Segment v2.1 R5752, a freely available software for medical image analysis (https://medviso.com/segment/). Described in Engblom et al. (2016) is the algorithm for infarct quantification from which we attained all ischemic measurements. A complete segmentation had all relevant slices for a given patient scan segmented into the endo- and epicardia for both the LV and RV, as well as potential ischemic tissue (Figure 1A). After segmentation of all slices, the extracted ventricular heart geometry for a given patient scan could be visualized as a 3D model as viewed in Figure 1C. All segmentation results were saved as binary MATLAB files (.mat extension).

#### 2.1.3. Slice Alignment and Surface Generation

Surface generation of segmented regions for creation of finite element models relies upon inter-slice registration for correct alignment, to remove patient motion artifacts. Marciniak et al. have previously described these methods in detail (Marciniak, 2017; Marciniak et al., 2017). The post-adjusted data was then extracted and converted into four separate surfaces (LV and RV endocardia, LV and biventricular epicardia). Surfaces were created using the Visualization Toolkit (VTK) (Schroeder et al., 2006). Ischemic points were converted into a surface using VTK and Insight Segmentation and Registration Toolkit (ITK) (Yoo et al., 2002). Surfaces were visualized in Paraview (Ayachit, 2015). All surfaces were stored as .vtk files.

#### 2.1.4. Finite Element Model Generation

The creation of 3D models based on generated surfaces was attained using gmsh (Geuzaine and Remacle, 2009). Mesh generation included three surfaces: the LV and RV endocardium and the biventricular epicardia as well as tetrahedral mesh of the ventricular myocardium and ischemic tissue. Following successful mesh generation, gmsh model output files were converted for use by the simulation software openCARP (Plank et al., 2021). The heterogeneous ischemic regions were incorporated into the ventricular mesh, first by generating volume and surface finite element meshes from the ischemic surface previously generated. Next, the ischemic volume was divided into numbered, layered regions representing a gradient of ischemic injury, with severity increasing toward the center of the damaged region. Regions were assigned based on distance from the outer surface of ischemic tissue using a scikit-learn Nearest Neighbors algorithm in Python (Pedregosa et al., 2011). For the baseline population, the number of regions for each model was between 10 and 27, depending on ischemia size. Finally, each point of ischemic volume and its corresponding region was mapped to the parent heart model. We additionally incorporated, tested, and implemented a node reordering optimization scheme for each resultant model to minimize eventual simulation times. Computation of rule-based myocardial fiber orientation was completed using the algorithm described in Bayer et al. (2012). Fibers are visualized in Figure 1B.

#### 2.1.5. Automation

The majority of the described pipeline is automated, excepting manual MRI segmentation, which takes about 15 min when completed by trained personnel. Once complete, the segmented binary .mat file can be input directly into the pipeline, resulting in the output of a personalized finite element heart model, including injured tissue, ready for use in simulations and further analysis. The pipeline is available to the public via GitHub at https://github.com/vildenst/3D-heart-models; the repository includes detailed installation and running instructions and offers access to all necessary software.

#### 2.1.6. Resultant Baseline Patient Models

Each of the 30 patient models in the baseline patient population is represented in Figure 1C; with healthy myocardium in red and the ischemic region in blue.

#### 2.1.7. Creating an Augmented Population of Patient Hearts

The MRI-based modeling pipeline described in previous sections was used to create several additional patient-geometry-based models. Ischemic volume could be effectively decreased from the baseline model, which incorporated the image-based ischemic tissue divided into several layered regions. In each patient heart, ischemic sizes were reduced by 1, 2, 5 and 10 layered regions to create four novel patient hearts with smaller ischemic sizes. Ischemic volume was reduced by defining the outer layers as electrophysiologically normal tissue, while a gradient from normal to fully ischemic tissue was used for the remaining inner layers. This process resulted in 99 additional, novel ischemic ventricular geometries derived from the original 30 patient hearts (the baseline population), resulting in a total of 129 ventricular models (the augmented population).

### 2.2. Electrophysiological Simulations and Determination of Arrhythmic Vulnerability

#### 2.2.1. Parameters Defining Conductivity and Electrophysiology

The ten Tusscher model represented healthy ventricular cell membrane electrophysiology (ten Tusscher and Panfilov, 2006), while damaged tissue in the ischemic region was modeled by alteration of ionic conductances as well reduced tissue conductivity in both the transverse and longitudinal directions as given below. Furthermore, we modeled ischemic regions as graded, with damage of increasing severity toward the center (Tomaselli and Zipes, 2004).

Presented in Supplementary Tables 1, 2 are the parameter values used for healthy tissue and ischemic regions, respectively (ten Tusscher and Panfilov, 2006; Kazbanov et al., 2014). All values are based on those used in a previous 3D model of human ventricular fibrillation (Kazbanov et al., 2014). Supplementary Table 1 gives parameter settings of the ten Tusscher model corresponding to a steep APD restitution slope of 1.8, increasing vulnerability to reentry. Supplementary Table 2 shows the example values for a five-layer ischemic region. The ischemic tissue was subdivided into 50% outer and 50% inner layer. This distribution was chosen as a large ischemic border zone has been shown to be pro-arrhythmogenic (Heidary et al., 2010). The innermost 50% of the ischemic tissue were modeled with 30% reduction in the INa and ICaL currents; while the outermost 50% were modeled with a 20% reduction in both currents compared to the healthy values. Extracellular potassium concentration was increased linearly from 7.5 to 10 mM from the outermost to the innermost ischemic regions. To further increase the arrhythmogenecity of the ischemic tissue, fATP was set to 0.0049 similar to what has been done previously (Ferrero et al., 2003). The resulting action potential traces are shown in Supplementary Figure 1.

Supplementary Table 3 references the tissue conductivities used for both healthy and ischemic tissue (Kléber et al., 1986; Poelzing et al., 2004; Akar et al., 2007; Hooks et al., 2007; Weiss et al., 2007; Clayton and Panfilov, 2008; Arevalo et al., 2016). Healthy conductivities have the same values as used previously (Arevalo et al., 2016) and ischemic conductivities have been reduced by 40% to model conduction slowing due to ischemia (Kléber et al., 1986; Akar et al., 2007; Jie and Trayanova, 2010).

#### 2.2.2. Pacing Site Selection and Vulnerability Simulation Protocol

**Figure 2:**
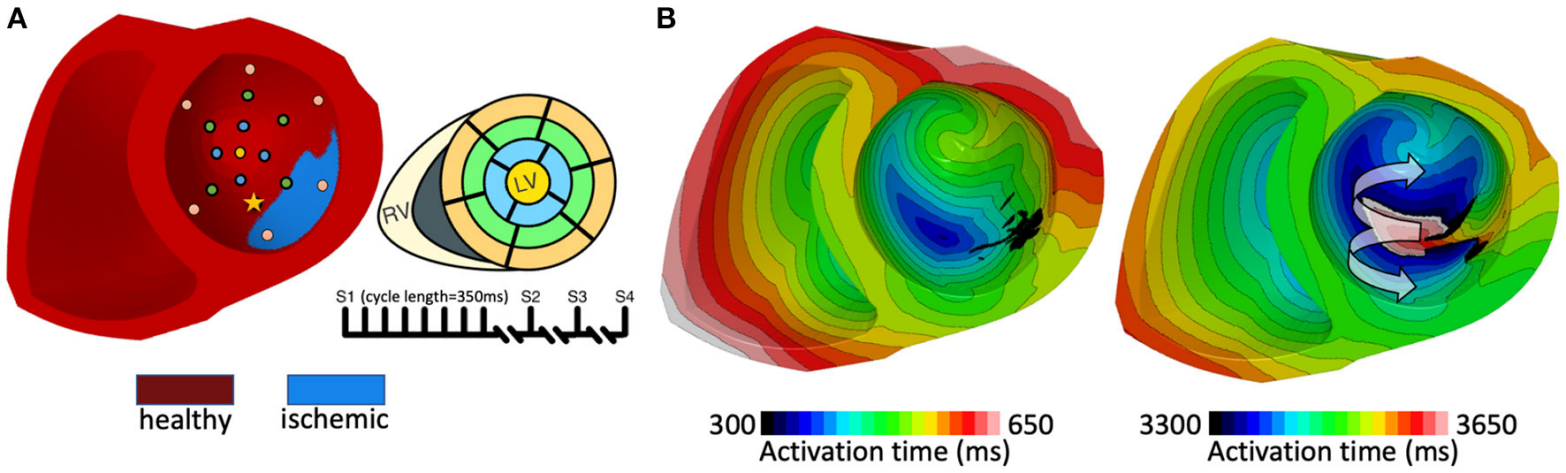
**(A)** Pacing induced arrhythmia protocol. The figure shows pacing sites on the myocardium (left), a schematic of corresponding AHA segments (top right) and pacing intervals (bottom right). Each stimulus had a duration of 10 ms, current amplitude of 100 uA/cm^2^ and electrode volume of 1 mm^3^. **(B)** Activation maps during a pacing train that resulted in induction of a sustained reentrant circuit. The stimulus was delivered near the ischemic border. Black region denote myocardium located deep within the ischemic tissue that did not excite due to the severity of the remodeling.

A simulated pacing protocol similar to standard clinical procedures triggering potential arrhythmic behavior was employed, as described previously (Cheng et al., 2013; Arevalo et al., 2016). Seventeen evenly distributed pacing sites in the LV, as based on the standard defined by the American Heart Association (AHA), were automatically selected as based on model orientation. As for other methods, this is available in the GitHub repository. Briefly, to each of these 17 LV pacing sites for each model, five pacing stimuli (S1) were delivered with a cycle length of 350 ms, followed by an S2 stimulus 200 ms following. If no arrhythmic behavior were detected, the S1-S2 period would be shortened by 10 ms intervals until there were reentrant circuits identified or until S2 failed to propagate. If the latter were the case, an S3 stimulus would be delivered 250 ms after the last successful S2, following the same procedure. Finally, an additional S4 stimulus would be delivered after 250 ms if no reentry were detected, following the same protocol as the S2 and S3 stimuli. Figure 2A illustrates the protocol described. Simulations were run for 2,000 ms following each delivered stimulus to detect potential arrhythmic activity, with outcomes defined as no reentry (NR), unsustained reentry (UR) or sustained reentry (R) (Figure 2B). The software used for simulations in this study is the open Cardiac Arrhythmia Research Package (openCARP) (Plank et al., 2021). All simulations were ran using 24 cores and 4G memory per CPU.

### 2.3. Arrhythmia Risk Classification Models

**Figure 3:**
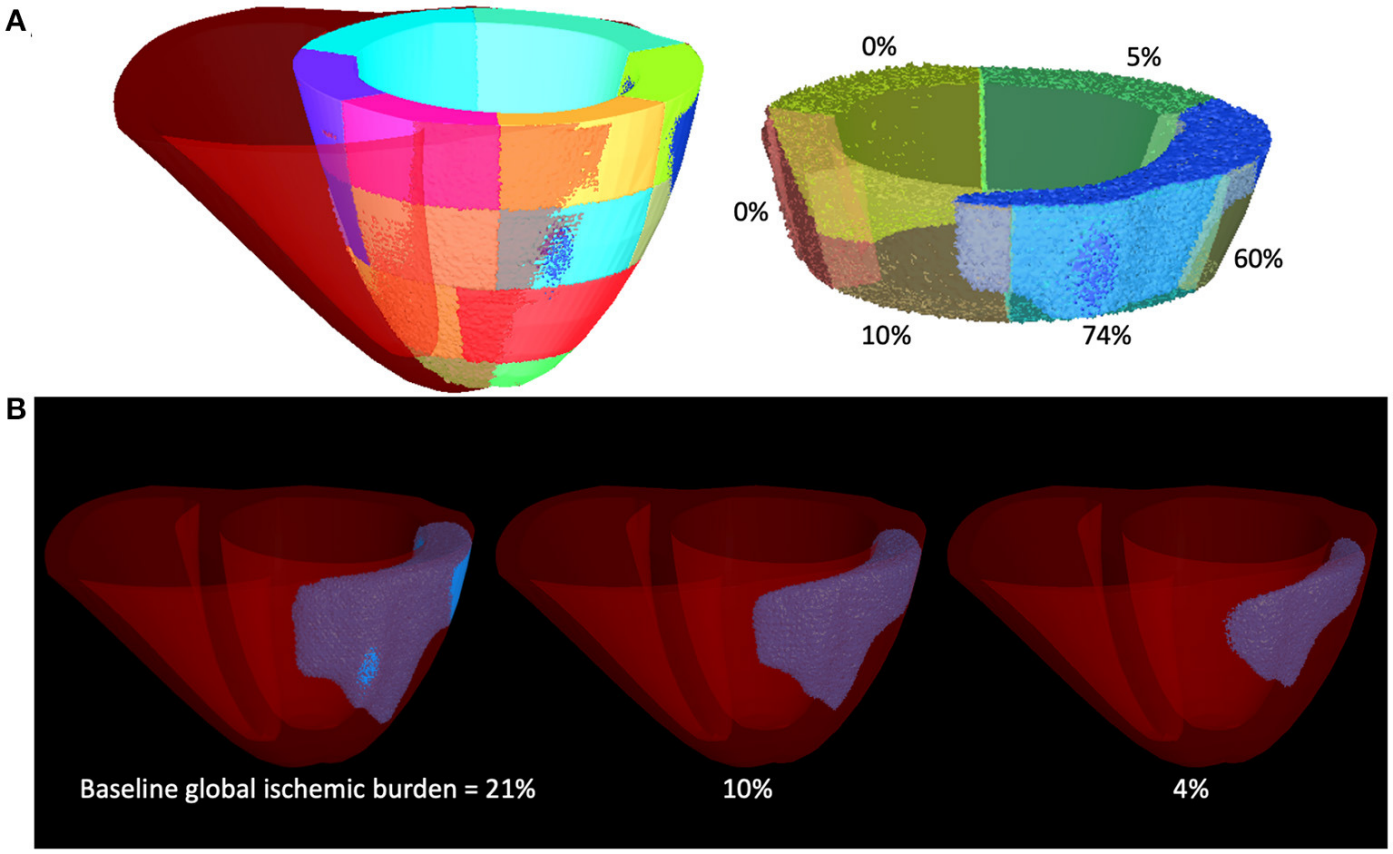
**(A)** Left to right: 3D representation of an exemplar patient model of the same 17-segment mapping, a medial slice of the same patient heart model showing accompanying percentages of segment-specific ischemic burden. **(B)** Left to right: the same heart with semi-transparent LV, showing the global ischemic burden in the baseline model, and this global burden reduced to 10 and then 4% to create two additional patient models for the augmented population. Both segment-specific and global myocardial volume and ischemic burdens were used as features for machine learning models in this study.

We assessed the ability of machine learning classification algorithms to correctly classify virtual patient arrhythmia risk (R and UR correspond to arrhythmia while NR corresponds to no arrhythmia) as based on simple virtual patient model-derived features. In each patient model (baseline plus augmented), global ischemia volume and global ventricular volume was measured, and for each of the 17 AHA LV segments the ischemic percentage and tissue volume was measured (4 features, Figures 3A,B).

#### 2.3.1. Machine Learning Algorithms

We investigated the performance of seven machine learning classification algorithms (ML models): K-nearest neighbors (knn), Gaussian support vector machine (SVM), logistic regression, decision tree (tree), xgboost, and 3- and 4-hidden layer multilayer perceptrons (feed-forward neural networks, 3-hl NN and 4-hl NN, respectively).

For all ML models, a chosen data set was shuffled randomly and split into train and test sets. The train and test sets were further individually standardized prior to model training and post-run model performance evaluation via the test set: the population mean was first subtracted then divided by standard deviation. Each model was trained on 70% of randomly selected segments and the remaining 30% of data (test set) was used for validation. This procedure was repeated for 100 runs of each ML model on each data set (both baseline and baseline + augmented).

#### 2.3.2. Model Implementation

k-nearest neighbors was implemented using sklearn's KNeighborsClassifier (k was set to 5). Support vector machine (SVM) was implemented using sklearn's SVC (C was set to 2). Logistic regression was implemented using sklearn's LogisticRegression. Tree was implemented using sklearn's DecisionTreeClassifier (max depth was set to 3). Xgboost was implemented using xgboost's XGBClassifier. 3-hl NN and 4-hl NN were implemented using Keras sequential (an API built on tensorflow). 3-hl NN used 32, 17 and 8 nodes, respectively, in each layer. Activation functions were the rectifier linear unit (ReLU) on hidden layers and the normalized exponential function (softmax) on output. Batch normalization was applied between each layer. The learning rate schedule was exponential decay with an initial learning rate of 0.01, with decay steps set to 100,000 and the decay rate to 0.9. We employed the gradient-based optimization methods RMSProp with zero momentum during training, as well as categorical crossentropy as the loss function. Twenty-five epochs, batch size = 20. 4-hl NN employed the same implementation as 3-hl NN, but with an additional layer of 8 nodes at its end.

Other than specified, default parameter values from sklearn, xgboost, and Keras sequential were used.

#### 2.3.3. Model Performance Assessment

Accuracy, equal to the number of correct predictions divided by the number of all predictions for the test data, was computed for all ML models for both baseline and augmented data sets. Precision was also calculated at a threshold of 0.5 for all ML models for both baseline and augmented data sets, to determine the proportion of positive arrhythmia identifications that were actually correct, defined as $precision=\frac{TP}{TP+FP}$. Model sensitivity (also known as *recall*), defined as $sensitivity=\frac{TP}{TP+FN}$ was additionally calculated at a threshold of 0.5 for ML model results on both baseline and augmented patient population results, where TP is the number of true positives, FP the number of false positives, and FN the number of false negatives. Average precision, a weighted mean of model precision for multiple thresholds, was also calculated for all ML models for both baseline and augmented data sets.

Receiver operating characteristic (ROC) curves were calculated for all models for both baseline and augmented population results (Melo, 2013b). The Area Under the ROC curve (AUC) was also calculated for all ML model results (Melo, 2013a).

P-values were calculated using a t-test to test whether per-model prediction accuracy improved when including the augmented patient population's simulated arrhythmia outcomes, as well as an F-test to test whether the per-model variance was smaller when including augmented population results.

## 3. Results

### 3.1. Arrhythmic Vulnerability in Baseline and Augmented Populations

In the baseline patient model population, 17 segments in 30 patients were evaluated for global arrhythmic vulnerability. Of these 510 segments, arrhythmia appeared in 111 segments during the protocol (no arrhythmia in 399; a ratio of 0.218). In the augmented patient model population, 17 segments in 129 total patient models were evaluated. Of these 2,193 segments, global arrhythmia appeared in 285 segments (no arrhythmia in 1,908; ratio 0.130) during our protocol. A summary of the results are given in Table 1.

**Table 1:** Summary of arrhythmia simulation results.

	**Baseline models**	**Augmented models**			
**no. of layers removed**	**0**	**1**	**2**	**5**	**10**
Total no. of patients	30	25	25	25	24
Mean global ischemia %	10.80	10.34	9.15	6.10	2.60
SD global ischemia %	10.54	8.65	7.86	5.62	2.77
no. of patients with reentry	17	15	10	8	3
% of patients with reentry	56.67	60.00	40.00	32.00	12.50
% of segments with reentry	21.76	18.82	13.41	7.53	1.18
Mean no. of reentries per model	3.70	3.20	2.28	1.28	0.21
SD no. of reentries per model	4.86	4.51	3.93	2.54	0.59

*Columns represent the baseline and augmented populations, where ischemic volumes are reduced by 1, 2, 5, and 10 layers (from left to right). Patients without ischemia were not further modified*.

The full set of results of the virtual vulnerability protocol for all patient models as specified in Materials and Methods can be found in Supplementary Table 4. Supplementary Table 5 summarizes the differences between the arrhythmic and non-arrhythmic groups. In general, hearts with larger global ischemic volumes were more inducible after the pacing protocol. Additonally, pacing from segments with larger percentage of ischemic tissue were also more likely to induce arrhythmia (Oliveira et al., 2018; Martinez-Navarro et al., 2019, 2021). This result suggests a mechanistic link between location of pacing site and arrhythmia inducibility in post-MI patients. These results are consistent with other studies that have shown that ectopic beats originating from the borders of ischemic tissue are more likely to result in wavebreak and reentry formation. Additionally, a positive correlation between ischemic volume and vulnerability to arrhythmia has been widely reported in the literature (Rubenstein et al., 2008; Klem et al., 2012).

### 3.2. Performance of Arrhythmic Risk Assessment Models

Segment-specific myocardial volume and segment-specific ischemic percentage as well as total myocardial volume and total estimated ischemic volume were calculated for each patient model in the baseline and augmented populations as detailed in Methods and Materials. Statistics on these features as well as associated arrhythmia outcomes can be found in Supplementary Table 5 (model input statistics).

Accuracy of all ML models trained and tested on data from both the baseline and augmented populations is shown in Table 2. For each of the seven ML models tested, mean predictive accuracy improved and accuracy standard deviation decreased when employing data from the augmented patient population. For all models trained and tested on data from the baseline patient population alone, SVM and logistic regression performed best in terms of mean accuracy (0.86; results among models ranged from 0.83 to 0.86). When using results from the augmented patient population, all ML models improved in accuracy (to 0.88 to 0.89; accuracy and variance of accuracy among all model trials between the baseline and augmented populations was statistically significant; *p*-values shown in Supplementary Table 8. Notably, 3- and 4-hl NN matched the performance of logistic regression, all performing best when considering the augmented population results.

**Table 2:** Results.

**Model**	**Mean**	**Standard deviation**	**Max**	**Min**
k-nearest neighbors, baseline	0.84386	0.02710	0.90196	0.77778
k-nearest neighbors, augmented	0.89468	0.00986	0.92553	0.87082
Support vector machine, baseline	0.86059	0.02358	0.92157	0.78431
Support vector machine, augmented	0.89453	0.01035	0.92097	0.87082
Logistic regression, baseline	0.86052	0.02342	0.92157	0.79085
Logistic regression, augmented	0.89749	0.01006	0.92097	0.87538
Decision tree, baseline	0.84261	0.02319	0.88889	0.78431
Decision tree, augmented	0.88868	0.01121	0.91489	0.86626
Xgboost, baseline	0.83118	0.02629	0.90196	0.73203
Xgboost, augmented	0.88722	0.00973	0.90881	0.86018
3 hidden layer neural network, baseline	0.84366	0.02785	0.92157	0.76471
3 hidden layer neural network, augmented	0.89353	0.01175	0.92401	0.86778
4 hidden layer neural network, baseline	0.84523	0.03018	0.90196	0.73856
4 hidden layer neural network, augmented	0.89470	0.01197	0.92249	0.86930

*ML model accuracy for baseline and augmented populations*.

Average precision (AP) of all ML models trained and tested on data from both the baseline and augmented populations is shown in Table 3. AP stayed the same, or modestly increased, for all ML models tested, with the exception of *SVM*, which decreased slightly. Sensitivity and precision of all ML models trained and tested on data from both the baseline and augmented populations at a threshold of 0.5 is additionally shown in Supplementary Tables 6, 7, respectively.

**Table 3:** Results.

**Model**	**Mean**	**Standard deviation**	**Max**	**Min**
k-nearest neighbors, baseline	0.86121	0.03168	0.93125	0.78341
k-nearest neighbors, augmented	0.86063	0.02158	0.91206	0.80355
Support vector machine, baseline	0.89963	0.02689	0.95743	0.82969
Support vector machine, augmented	0.88938	0.01627	0.92553	0.83582
Logistic regression, baseline	0.90478	0.02278	0.95267	0.84192
Logistic regression, augmented	0.9187	0.01033	0.94464	0.88435
Decision tree, baseline	0.89213	0.02918	0.94562	0.79392
Decision tree, augmented	0.89281	0.01768	0.9265	0.83708
Xgboost, baseline	0.87596	0.02704	0.9257	0.77737
Xgboost, augmented	0.90071	0.01106	0.91898	0.86177
3 hidden layer neural network, baseline	0.89673	0.02518	0.95611	0.79616
3 hidden layer neural network, augmented	0.91565	0.01147	0.94856	0.88724
4 hidden layer neural network, baseline	0.90069	0.02378	0.95003	0.82399
4 hidden layer neural network, augmented	0.91448	0.01316	0.94674	0.87139

*ML model average precision for baseline and augmented populations*.

**Figure 4:**
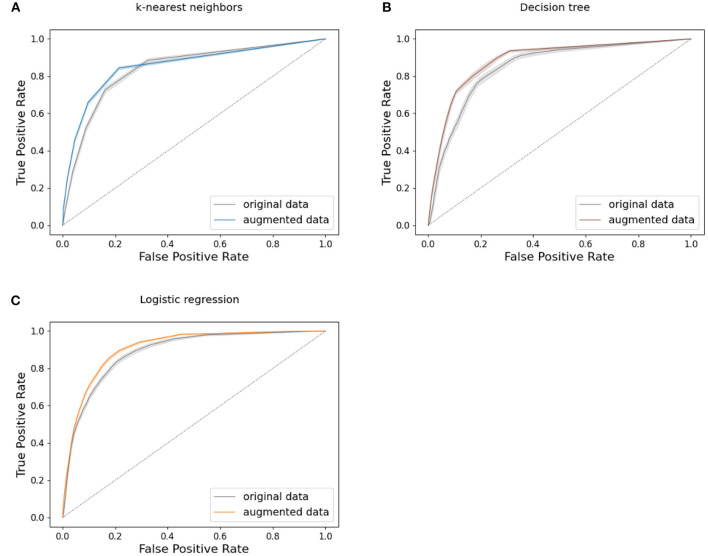
Machine learning model performance: ROC curves with 95% confidence interval for **(A)** k-nearest neighbors, **(B)** decision tree, and **(C)** logistic regression, comparing models trained on augmented and baseline population. True positive rate = *TP*/(*TP* + *FN*), false positive rate = *FP*/(*FP* + *TN*).

**Figure 5:**
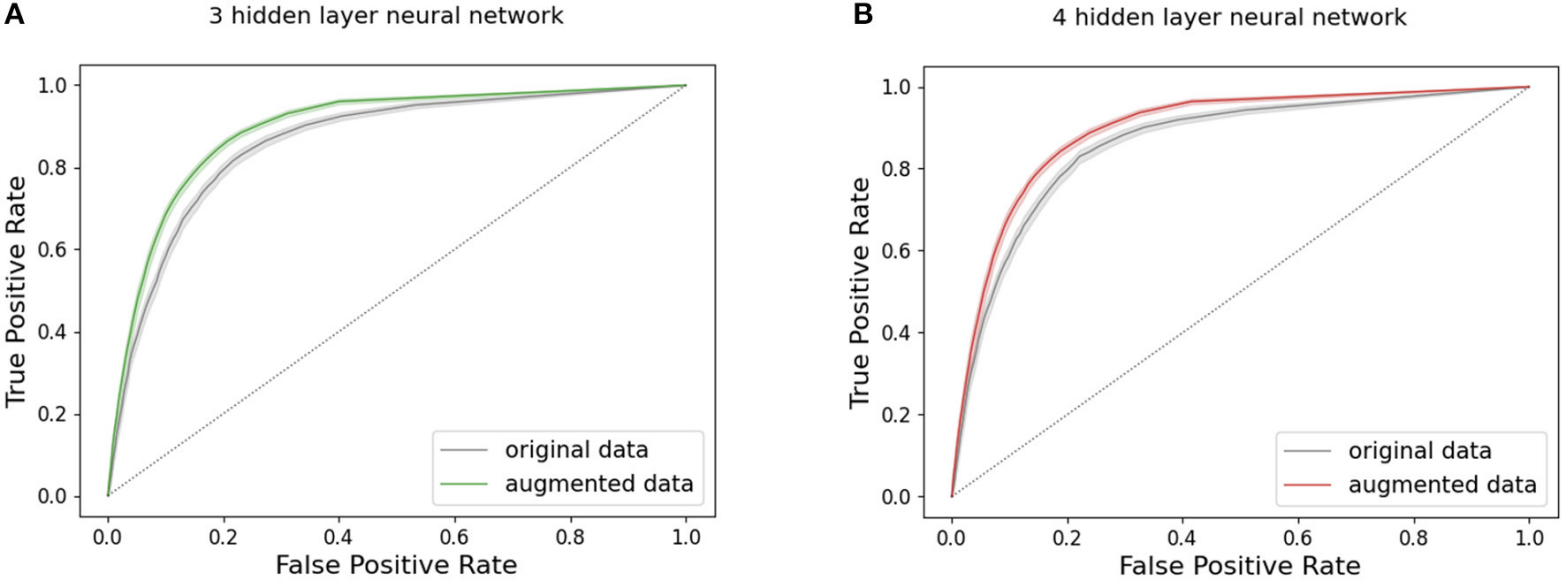
Artificial neural network performance: ROC curves with 95% confidence interval for **(A)** 3 and **(B)** 4 hidden layer feedforward neural network, comparing models trained on augmented and baseline population. True positive rate = *TP*/(*TP* + *FN*), false positive rate = *FP*/(*FP* + *TN*).

Figures 4, 5 present the ROC curves for the highest-performing models tested in both the baseline and augmented populations, knn, decision tree, and logistic regression are shown in Figure 4, while neural network ROC curves are presented in Figure 5. Supplementary Figure 2 shows results for SVM and xgboost. Corresponding AUC for all ML models trained and tested on data from both the baseline and augmented populations is shown in Table 4. While differences among models are relatively modest, logistic regression, 3-hl NN, and 4-hl NN performed similarly best in class, with confidence intervals as shown in Figures 4B, 5A,B.

**Table 4:** Results.

**Model**	**Mean**	**Standard deviation**	**Max**	**Min**
k-nearest neighbors, baseline	0.86121	0.03168	0.93125	0.78341
k-nearest neighbors, augmented	0.86063	0.02158	0.91206	0.80355
Support vector machine, baseline	0.89963	0.02689	0.95743	0.82969
Support vector machine, augmented	0.88938	0.01627	0.92553	0.83582
Logistic regression, baseline	0.90478	0.02278	0.95267	0.84192
Logistic regression, augmented	0.9187	0.01033	0.94464	0.88435
Decision tree, baseline	0.85995	0.03819	0.92412	0.74988
Decision tree, augmented	0.89281	0.01768	0.9265	0.83708
Xgboost, baseline	0.87596	0.02704	0.9257	0.77737
Xgboost, augmented	0.90071	0.01106	0.91898	0.86177
3 hidden layer neural network, baseline	0.89673	0.02518	0.95611	0.79616
3 hidden layer neural network, augmented	0.91565	0.01147	0.94856	0.88724
4 hidden layer neural network, baseline	0.90069	0.02378	0.95003	0.82399
4 hidden layer neural network, augmented	0.91448	0.01316	0.94674	0.87139

*ML model AUC for baseline and augmented populations*.

## 4. Discussion

### 4.1. Summary of Study and Findings

Here, we have presented a combined *in silico* and machine learning methodology to assess risk for dangerous arrhythmia in post-infarct patients. We have aimed to briefly demonstrate that simulation-supported data augmentation can improve prediction models and overall accuracy for arrhythmia risk assessment. Briefly, we used a semi-automated image-based patient-specific modeling and simulation pipeline to create both baseline and augmented patient populations, and assessed vulnerability to reentry in both populations via a virtual programmed stimulation protocol. We then calculated specific geometric features in all patient models, and trained seven machine learning algorithms (3 classification, 1 clustering, and 2 neural networks) to predict arrhythmia outcome directly from these geometric patient model features alone.

We found that this combined approach can provide insight with high accuracy, rapidly and efficiently, with accuracy ranging from 83 to 86% for all ML models for the baseline population. Furthermore, all ML models improved in accuracy to 88–89% (accuracy and accuracy variance was statistically significant; *p*-values shown in Supplementary Table 8) when using results from the augmented patient population, demonstrating that, particularly in the case of small cohorts and/or sparse patient data, simulation-supported data augmentation can be employed to further improve results of predictive machine learning models.

### 4.2. Comment on Model Explainability and Critical Features

Previous research has identified LGE volume (Klem et al., 2012) and LV mass (Haider et al., 1998) as predictors for sudden cardiac death. Because we implemented a decision tree as one of the ML models evaluated and this performed reasonably similar to other models, we were conveniently able to directly probe the decision-making in this algorithm to assess which feature(s) this model deemed as most-important for its decision making. Again, the hand-picked geometric features were: segment-specific myocardial volume and ischemic percentage, as well as total myocardial volume and total estimated ischemic volume, calculated for each patient model and segment in both the baseline and augmented populations. In both populations, the most important of the four input features tested was estimated total ischemic volume (Supplementary Figures 3, 4, respectively). However, in the augmented population, the other three features (total myocardial volume, segment-specific ischemic percentage, and segment-specific myocardial volume) were more important for decision making than in the baseline population. The decision trees for the baseline and augmented populations can be seen in Supplementary Figures 5, 6, respectively.

### 4.3. Biophysical Model-Based Data Creation and Augmentation: A Growing Body of Work

ML models have been utilized successfully and extensively in arrhythmia risk assessment (Feeny et al., 2020; Krittanawong et al., 2020) and in cardiovascular imaging, to diverse ends (Prakosa et al., 2013; Bernard et al., 2018; Sermesant et al., 2021). More recently, compound, explainable ML models have demonstrated improved risk prediction for ventricular arrhythmias as compared to traditional biomarkers (i.e., left ventricular ejection fraction, LVEF), as validated retrospectively in large clinical cohorts, (e.g., Ly et al., 2021). However, others in recent years have also pioneered the combination of biophysical modeling and ML approaches in arrhythmia risk assessment (Lamata, 2018). In cardiac electrophysiology and arrhythmias, applications include but are not limited to techniques for electrical mapping of the myocardium, research to uncover the basic mechanisms of arrhythmia, and arrhythmia treatment planning and management, as recently reviewed in Trayanova et al. (2021). A key utilization of biophysical model-enabled data creation in this space has been for feature augmentation to improve performance of learning schemes (Lozoya et al., 2019; Shade et al., 2020). Notably, Shade et al. (2020) used ML and personalized computational modeling in concert to accurately predict whether a patient was likely to experience AF recurrence following pulmonary vein isolation (PVI), using only pre-PVI LGE-MRI scans as input. This work shares some notable methodological similarities with the present study: only patient LGE-MRI were used as input for electrophysiological computational models, and the (baseline) patient cohort size was similar (32 vs. 0.30 in the present study). Also similarly, Shade et al. found reasonable predictive performance with traditional ML approaches and a simulation-augmented feature set. The divergence in the current study is that the input data was augmented via patient model population expansion, rather than introduction of additional features, e.g., from our electrophysiological simulations. Corrado et al. (2021) also recently demonstrated the use of a virtual patient cohort to assess risk for sustained atrial arrhythmia; an ML model (SVM) trained on local conduction velocity and action potential duration was able to accurately predict whether an arrhythmia would tether to that tissue region. In the present study, simulations also provided the ground-truth regarding patient vulnerability to arrhythmia, as required for this proof-of-concept in post-MI patients. In future work, we will indeed employ electrophysiological features from simulations themselves to assess how their incorporation improves and/or alters model performance.

### 4.4. Limitations and Future Work

Despite the successful proof-of-concept executed in this study, there are acknowledged limitations to this work. In order to expand the number of patient hearts in the augmented population, the ischemic tissue in each patient heart was reduced several times, as described in Methods and Materials. A naive approach was thus adopted as a first step and ischemic volume was not altered symmetrically, given practical limitations in terms of computational time and tractability for patient-specific biophysical simulations.

The patient population can be further augmented in several ways to explore the empirical role of class balance in classifier performance, as well as to create data of sufficient volume to explore the improved performance of vanilla NN and deep learning approaches e.g., convolutional neural networks. Approaches to be used for augmenting patient populations (the space of the patient-specific, image-derived geometries and concomitant features) include shape modeling approaches (Balaban et al., 2021) as well as generative adversarial networks (Gholami et al., 2018; Shaker et al., 2020). Next steps for this and related work research may include combining multi-organ systems for joint study (e.g., Banus et al., 2021), to both better constrain the parameter space of a personalized model and to subsequently capture plausible physiologically mechanisms.

Furthermore, we have employed LGE-MRI from patients 5 days post-MI and have considered the damaged tissue region as ischemic in the present study, rather than as an evolving necrotic/fibrotic scar region. It is known, however, that the initial region of ischemic injury evolves rapidly, spatially and functionally, and may change significantly by the time of imaging 5 days later (Anversa and Sonnenblick, 1990; Holmes et al., 1994; Ertl and Frantz, 2005; Geerse et al., 2009; Wan Ab Naim et al., 2020), introducing uncertainty into our assumptions regarding the modeling of damaged tissue.

Finally, to demonstrate potential clinical utility of the method, validation of trained ML models with e.g., paired clinical follow-up data for arrhythmia incidence for the non-augmented population would be critical. Simulation results as presented here and real clinical scenarios may be quite different; for instance, the overall clinical arrhythmia rate may differ between specific patient groups and from *in silico* incidence, and should be taken into consideration. Presently, while comparison between simulation results here and clinical data has not been possible due to lack of appropriate data, appropriate follow-up data and model validation is ultimately crucial for the method's translational utility.

## Data Availability Statement

The raw simulation data supporting the conclusions of this article will be made available by the authors, without undue reservation.

## Author Contributions

MM co-designed the study, wrote the manuscript, co-directed student work, and co-designed figures. LM augmented the image-based patient models and ran simulations. JU trained all machine learning models and conducted statistical analyses. PF designed and implemented initial machine learning models for the baseline population. VS constructed baseline patient models, ran simulations, and implemented image-based modeling pipeline. CG conducted clinical measurements in patient study. RJ conducted clinical measurements in patient study. KA and NV conducted the clinical MRI. TE participated in patient study. TJ co-designed and contributed to clinical study. JT-H co-designed and directed patient study. VN co-designed and consulted on initial study. HA co-designed the study, directed student work, edited the manuscript, and co-designed figures. All authors reviewed the final manuscript.

## Funding

HA and MM Simula Research Laboratory, Ministry of Research and Education of Norway, NO. LM and JU Research Council of Norway (303178), European, and/or other funding source. ProCardio Norwegian Center for Research-Based Innovation, Research Council of Norway, NO. MI-RISK Novo Nordisk Interdisciplinary Synergy Grant, Novo Nordisk Fond, DK.

## Conflict of Interest

The authors declare that the research was conducted in the absence of any commercial or financial relationships that could be construed as a potential conflict of interest.

## Publisher's Note

All claims expressed in this article are solely those of the authors and do not necessarily represent those of their affiliated organizations, or those of the publisher, the editors and the reviewers. Any product that may be evaluated in this article, or claim that may be made by its manufacturer, is not guaranteed or endorsed by the publisher.
