# Supplementary material for: Combined In-silico and Machine Learning Approaches Toward Predicting Arrhythmic Risk in Post-infarction Patients
Source: Front Physiol. 2021 Nov 8;12:745349. doi: 10.3389/fphys.2021.745349 (PMC8606551; doi:10.3389/fphys.2021.745349)
Supplement: Supplementary file 1 [file Data_Sheet_1.pdf]

Supplementary Materials

Table S1: Supplementary Methods: Conductances (nS/pF) for healthy myocardium corresponding to the Ten Tusscher model with a steep restitution slope of 1.8. Remaining parameters are as given in the original Ten Tusscher model.

	$g_{\mathbf{Kr}}$	$g_{\mathbf{Ks}}$	$g_{\mathbf{pCa}}$	$g_{\mathbf{pK}}$	$[\mathbf{K}^+]_o$	$f_{\mathbf{ATP}}$
Value	0.172	0.441	0.867	0.00219	5.4	0

Table S2: Supplementary Methods: Conductances (percent of base values), $[K^+]_o$ (mM) and f_{ATP} (fraction of open I_{KATP} channels) for a five-layer ischemic region. All other parameters are the same as in healthy myocardium.

	$g_{\mathbf{Na}}$	$g_{\mathbf{CaL}}$	$[\mathbf{K}^+]_o$	$f_{\mathbf{ATP}}$
Outer ischemic region	80%	80%	7.5	0.0049
	80%	80%	8	0.0049
	70%	70%	8.5	0.0049
	70%	70%	9	0.0049
Inner ischemic region	70%	70%	10	0.0049

Table S3: Supplementary Methods: Conductivity values (S/m) for healthy and ischemic tissue.

	**Healthy tissue**	**Ischemic tissue**
Longitudinal direction	0.255	0.153
Transverse direction	0.0775	0.0465

Table S4: Supplementary Results: Arrhythmic vulnerability in patient models. Each column gives simulation results for a single model with the given ischemic percentage, i.e. percentage of left ventricular tissue with ischemic properties. The second column refers to the original model, while the third to sixth columns refer to augmented models. Results are classified as NR (no reentry), UR (unsustained reentry) or R (sustained reentry) during S1, S2, S3 or S4 of the pacing protocol.

Patient 1

**Pacing**	**Ischemia percentage**				
**Site**	**3.62%**	**2.57%**	**2.06%**	**1.19%**	**0.26%**
site1	NR	NR	NR	NR	NR
site2	NR	NR	NR	NR	NR
site3	NR	NR	NR	NR	NR
site4	NR	NR	NR	NR	NR
site5	NR	NR	NR	NR	NR
site6	NR	NR	NR	NR	NR
site7	NR	NR	NR	NR	NR
site8	NR	NR	NR	NR	NR
site9	NR	NR	NR	NR	NR
site10	NR	NR	NR	NR	NR
site11	NR	NR	NR	NR	NR
site12	NR	NR	NR	NR	NR
site13	NR	NR	NR	NR	NR
site14	NR	NR	NR	NR	NR
site15	NR	NR	NR	NR	NR
site16	NR	NR	NR	NR	NR
site17	NR	NR	NR	NR	NR

Supplementary Materials

Patient 2

**Pacing Site**	**Ischemia percentage**				
	**9.79%**	**7.46%**	**6.56%**	**4.40%**	**2.01%**
site1	NR	NR	NR	NR	NR
site2	NR	NR	NR	NR	NR
site3	NR	NR	NR	NR	NR
site4	NR	NR	NR	NR	NR
site5	NR	Failed stimulus	Failed stimulus	NR	NR
site6	NR	NR	NR	NR	NR
site7	NR	NR	NR	NR	NR
site8	NR	NR	NR	NR	NR
site9	NR	NR	NR	NR	NR
site10	NR	NR	NR	NR	NR
site11	NR	NR	NR	NR	NR
site12	NR	NR	NR	NR	NR
site13	NR	NR	NR	NR	NR
site14	NR	NR	NR	NR	NR
site15	NR	UR at s4	NR	NR	NR
site16	NR	NR	NR	NR	NR
site17	NR	NR	NR	NR	NR

Patient 3

**Pacing Site**	**Ischemia percentage**				
	**10.16%**	**7.84%**	**6.85%**	**4.26%**	**1.29%**
site1	NR	NR	NR	NR	NR
site2	UR at s3	NR	NR	NR	NR
site3	UR at s3	NR	NR	NR	NR
site4	R at s2	UR at s3	NR	NR	NR
site5	NR	UR at s3	R at s4	NR	NR
site6	NR	NR	NR	NR	NR
site7	NR	NR	NR	NR	NR
site8	NR	NR	NR	NR	NR
site9	UR at s4	R at s3	R at s4	NR	NR
site10	R at s2	R at s4	NR	NR	NR
site11	UR at s4	NR	NR	NR	NR
site12	NR	NR	NR	NR	NR
site13	NR	NR	NR	NR	NR
site14	NR	NR	NR	NR	NR
site15	NR	NR	NR	NR	NR
site16	R at s4	NR	NR	NR	NR
site17	NR	R at s4	NR	NR	NR

Patient 4

Pacing Site	Ischemia percentage 0.33%	0.23%	0.18%	0.09%	0.01%
site1	NR	NR	NR	NR	NR
site2	NR	NR	NR	NR	NR
site3	NR	NR	NR	NR	NR
site4	NR	NR	NR	NR	NR
site5	NR	NR	NR	NR	NR
site6	NR	NR	NR	NR	NR
site7	NR	NR	NR	NR	NR
site8	NR	NR	NR	NR	NR
site9	NR	NR	NR	NR	NR
site10	NR	NR	NR	NR	NR
site11	NR	NR	NR	NR	NR
site12	NR	NR	NR	NR	NR
site13	NR	NR	NR	NR	NR
site14	NR	NR	NR	NR	NR
site15	NR	NR	NR	NR	NR
site16	NR	NR	NR	NR	NR
site17	NR	NR	NR	NR	NR

Patient 5

Pacing Site	Ischemia percentage 14.56%	11.31 %	9.90 %	6.23 %	1.98 %
site1	NR	NR	NR	NR	NR
site2	R at s2	R at s2	NR	R at s3	NR
site3	R at s3	R at s3	R at s4	NR	NR
site4	NR	R at s4	R at s4	NR	NR
site5	NR	NR	NR	NR	NR
site6	NR	NR	NR	NR	NR
site7	NR	NR	NR	NR	NR
site8	R at s3	R at s2	R at s2	R at s4	NR
site9	NR	NR	NR	NR	NR
site10	NR	NR	NR	NR	NR
site11	NR	NR	NR	NR	NR
site12	UR at s4	NR	NR	NR	NR
site13	NR	NR	NR	NR	NR
site14	UR at s4	NR	NR	NR	NR
site15	NR	NR	NR	NR	NR
site16	UR at s4	NR	NR	NR	NR
site17	NR	NR	NR	NR	NR

Patient 6

**Pacing Site**	**Ischemia percentage** 8.43 %	6.31%	5.47%	3.49%	1.21%
site1	NR	NR	NR	NR	NR
site2	NR	NR	NR	NR	NR
site3	NR	NR	UR at s4	NR	NR
site4	NR	NR	NR	NR	NR
site5	UR at s4	NR	NR	NR	NR
site6	UR at s5	NR	NR	NR	NR
site7	NR	NR	NR	NR	NR
site8	NR	NR	NR	NR	NR
site9	NR	NR	NR	NR	NR
site10	NR	NR	NR	NR	NR
site11	NR	NR	NR	NR	NR
site12	NR	NR	NR	NR	NR
site13	NR	UR at s4	NR	NR	NR
site14	NR	NR	NR	NR	NR
site15	NR	NR	NR	NR	NR
site16	NR	NR	NR	NR	NR

Patient 7

**Pacing Site**	**Ischemia percentage** 33.69 %	28.61 %	25.91%	18.24%	7.91%
site1	R at s2	UR at s2	UR at s2	UR at s3	NR
site2	UR at s2	UR at s2	UR at s2	UR at s2	UR at s3
site3	UR at s4	UR at s4	UR at s3	NR	NR
site4	UR at s3	UR at s3	R at s4	UR at s4	NR
site5	UR at s4	UR at s4	R at s4	NR	NR
site6	R at s4	NR	NR	NR	NR
site7	No propagation	UR at s2	NR	UR at s3	NR
site8	UR at s4	UR at s4	UR at s4	NR	NR
site9	NR	NR	NR	NR	NR
site10	UR at s4	NR	UR at s3	UR at s3	NR
site11	R at s4	UR at s4	R at s4	R at s4	NR
site12	R at s4	UR at s3	R at s4	NR	NR
site13	R at s4	R at s4	NR	NR	NR
site14	NR	UR at s4	UR at s4	UR at s3	UR at s3
site15	R at s2	NR	NR	UR at s3	NR
site16	NR	NR	NR	NR	NR

Patient 8

**Pacing Site**	**Ischemia percentage 23.21%**	**18.26%**	**16.48%**	**11.75%**	**5.91%**
site1	NR	NR	NR	NR	NR
site2	NR	NR	NR	NR	NR
site3	NR	NR	NR	NR	NR
site4	NR	NR	NR	NR	NR
site5	NR	NR	NR	NR	NR
site6	NR	NR	NR	NR	NR
site7	UR at s3	UR at s4	NR	NR	NR
site8	NR	NR	NR	NR	NR
site9	NR	NR	NR	NR	NR
site10	NR	NR	NR	NR	NR
site11	UR at s3	NR	NR	NR	NR
site12	UR at s3	NR	NR	NR	NR
site13	UR at s4	NR	NR	NR	NR
site14	UR at s4	NR	NR	NR	NR
site15	NR	NR	NR	NR	NR
site16	UR at s3	NR	NR	NR	NR
site17	UR at s4	NR	NR	NR	NR

Patient 9

**Pacing Site**	**Ischemia percentage 0%**
site1	NR
site2	NR
site3	NR
site4	NR
site5	NR
site6	NR
site7	NR
site8	NR
site9	NR
site10	NR
site11	NR
site12	NR
site13	NR
site14	NR
site15	NR
site16	NR
site17	NR

Patient 10

**Pacing Site**	**Ischemia percentage 0%**
site1	NR
site2	NR
site3	NR
site4	NR
site5	NR
site6	NR
site7	NR
site8	NR
site9	NR
site10	NR
site11	NR
site12	NR
site13	NR
site14	NR
site15	NR
site16	NR
site17	NR

Patient 11

**Pacing Site**	**Ischemia percentage 0%**
site1	NR
site2	NR
site3	NR
site4	NR
site5	NR
site6	NR
site7	NR
site8	NR
site9	NR
site10	NR
site11	NR
site12	NR
site13	NR
site14	NR
site15	NR
site16	NR
site17	NR

Patient 12

Pacing Site	Ischemia percentage 31.42%	25.94%	23.17%	15.92%	7.10%
site1	UR at s4	UR at s4	NR	NR	NR
site2	UR at s4	UR at s4	UR at s4	NR	NR
site3	NR	UR at s4	NR	NR	NR
site4	UR at s4	UR at s3	UR at s4	UR at s4	NR
site5	NR	UR at s3	R at s4	UR at s4	NR
site6	NR	NR	NR	NR	NR
site7	UR at s4	UR at s2	UR at s4	NR	NR
site8	UR at s3	R at s4	R at s3	UR at s3	NR
site9	UR at s2	R at s2	UR at s4	UR at s4	NR
site10	UR at s3	UR at s3	UR at s4	UR at s4	NR
site11	UR at s4	R at s4	R at s4	NR	NR
site12	NR	UR at s4	UR at s4	NR	NR
site13	UR at s2	UR at s2	UR at s4	UR at s3	NR
site14	UR at s4	UR at s4	R at s4	R at s4	NR
site15	UR at s4	NR	NR	UR at s3	NR
site16	UR at s3	NR	UR at s3	NR	NR
site17	UR at s4	R at s2	R at s3	UR at s3	NR

Patient 13

Pacing Site	Ischemia percentage 21.33%	17.51%	15.59%	10.44%	4.03%
site1	NR	NR	NR	NR	NR
site2	UR at s4	UR at s4	NR	NR	NR
site3	UR at s4	UR at s3	NR	R at s4	NR
site4	UR at s3	R at s3	NR	R at s3	NR
site5	NR	UR at s4	NR	NR	UR at s2
site6	NR	UR at s4	R at s4	R at s4	NR
site7	NR	UR at s4	NR	NR	NR
site8	NR	NR	NR	NR	NR
site9	UR at s4	R at s4	NR	NR	NR
site10	R at s2	NR	UR at s2	UR at s3	NR
site11	NR	NR	NR	NR	UR at s3
site12	NR	NR	NR	NR	NR
site13	R at s4	UR at s4	NR	NR	NR
site14	UR at s3	R at s4	UR at s3	NR	NR
site15	NR	R at s4	NR	NR	NR
site16	NR	UR at s4	UR at s4	NR	NR
site17	UR at s4	UR at s4	UR at s4	R at s4	NR

Patient 14

**Pacing Site**	**Ischemia percentage 0%**
site1	NR
site2	NR
site3	NR
site4	NR
site5	NR
site6	NR
site7	NR
site8	NR
site9	NR
site10	NR
site11	NR
site12	NR
site13	NR
site14	NR
site15	NR
site16	NR
site17	NR

Patient 15

**Pacing**			**Ischemia percentage**		
**Site**	**7.49%**	**5.52%**	**4.63%**	**2.59%**	**0.49 %**
site1	NR	NR	NR	NR	NR
site2	NR	NR	NR	NR	NR
site3	NR	NR	NR	NR	NR
site4	NR	R at s2	NR	UR at s2	NR
site5	NR	NR	NR	NR	NR
site6	NR	NR	NR	NR	NR
site7	NR	NR	NR	NR	NR
site8	NR	NR	NR	NR	NR
site9	NR	NR	NR	NR	NR
site10	NR	NR	NR	NR	NR
site11	R at s4	NR	NR	NR	NR
site12	NR	NR	NR	NR	NR
site13	NR	NR	NR	NR	NR
site14	NR	NR	NR	NR	NR
site15	NR	NR	NR	NR	NR
site16	NR	NR	NR	NR	NR
site17	NR	NR	NR	NR	NR

Patient 16

**Pacing Site**	**Ischemia percentage**				
	**5.43 %**	**4.37%**	**3.83%**	**2.51%**	**1.02%**
site1	NR	NR	NR	NR	NR
site2	NR	NR	NR	NR	NR
site3	NR	NR	NR	NR	NR
site4	NR	NR	NR	NR	NR
site5	NR	NR	NR	NR	NR
site6	NR	NR	NR	NR	NR
site7	NR	NR	NR	NR	NR
site8	NR	NR	NR	NR	NR
site9	NR	NR	NR	NR	NR
site10	NR	NR	NR	NR	NR
site11	NR	NR	NR	NR	NR
site12	NR	NR	NR	NR	NR
site13	NR	NR	NR	NR	NR
site14	NR	NR	NR	NR	NR
site15	NR	NR	NR	NR	NR
site16	NR	NR	NR	NR	NR
site17	NR	NR	NR	NR	NR

Patient 17

**Pacing Site**	**Ischemia percentage**				
	**10.26%**	**7.88%**	**6.84%**	**4.27%**	**1.54%**
site1	NR	NR	NR	NR	NR
site2	NR	NR	NR	NR	NR
site3	NR	NR	NR	NR	NR
site4	NR	NR	NR	NR	NR
site5	UR at s2	UR at s2	NR	NR	NR
site6	NR	NR	NR	NR	NR
site7	NR	NR	NR	NR	NR
site8	NR	NR	NR	NR	NR
site9	NR	NR	NR	NR	NR
site10	R at s4	NR	NR	NR	NR
site11	NR	NR	NR	NR	NR
site12	NR	NR	NR	NR	NR
site13	NR	NR	NR	NR	NR
site14	NR	NR	NR	NR	NR
site15	NR	NR	NR	NR	NR
site16	NR	NR	NR	NR	NR
site17	NR	NR	NR	NR	NR

Patient 18

**Pacing Site**	**Ischemia percentage** 18.02%	14.17%	12.55%	8.29%	3.07 %
site1	UR at s4	NR	NR	NR	NR
site2	NR	NR	NR	NR	NR
site3	NR	UR at s4	UR at s4	NR	NR
site4	R at s4	NR	NR	NR	NR
site5	UR at s4	NR	NR	NR	NR
site6	NR	UR at s4	UR at s4	NR	NR
site7	UR at s4	NR	NR	NR	NR
site8	UR at s3	NR	NR	NR	NR
site9	NR	NR	NR	NR	NR
site10	UR at s4	UR at s4	UR at s4	UR at s4	NR
site11	NR	UR at s4	UR at s4	NR	NR
site12	NR	NR	NR	NR	NR
site13	UR at s4	UR at s3	UR at s3	NR	NR
site14	NR	NR	NR	NR	NR
site15	UR at s4	NR	NR	NR	NR
site16	UR at s4	NR	NR	NR	NR
site17	NR	NR	NR	NR	NR

Patient 19

**Pacing Site**	**Ischemia percentage** 0.09%	0.06%	0.05%	0.02%
site1	NR	NR	NR	NR
site2	NR	NR	NR	NR
site3	NR	NR	NR	NR
site4	NR	NR	NR	NR
site5	NR	NR	NR	NR
site6	NR	NR	NR	NR
site7	NR	NR	NR	NR
site8	NR	NR	NR	NR
site9	NR	NR	NR	NR
site10	NR	NR	NR	NR
site11	NR	NR	NR	NR
site12	NR	NR	NR	NR
site13	NR	NR	NR	NR
site14	NR	NR	NR	NR
site15	NR	NR	NR	NR
site16	NR	NR	NR	NR
site17	NR	NR	NR	NR

Patient 20

Pacing Site	Ischemia percentage 34.13 %	29.10%	26.66%	19.71%	10.62%
site1	UR at s2	UR at s4	UR at s4	NR	NR
site2	R at s4	NR	NR	NR	NR
site3	R at s4	UR at s4	UR at s4	NR	NR
site4	UR at s4	NR	NR	NR	NR
site5	R at s4	NR	NR	NR	NR
site6	R at s2	R at s3	R at s4	NR	NR
site7	UR at s3	R at s4	UR at s4	UR at s4	NR
site8	R at s4	NR	NR	NR	NR
site9	R at s3	NR	NR	NR	NR
site10	R at s3	NR	NR	NR	NR
site11	UR at s3	NR	NR	NR	NR
site12	NR	NR	UR at s4	UR at s4	NR
site13	R at s3	NR	NR	NR	NR
site14	R at s3	NR	NR	NR	NR
site15	R at s3	NR	NR	NR	NR
site16	R at s3	R at s4	NR	NR	NR
site17	R at s3	NR	NR	NR	NR

Patient 21

Pacing Site	Ischemia percentage 4.23%	3.11%	2.67%	1.69%	0.59%
site1	NR	NR	NR	NR	NR
site2	NR	NR	NR	NR	NR
site3	NR	NR	NR	NR	NR
site4	NR	NR	NR	NR	NR
site5	UR at s4	NR	NR	NR	NR
site6	NR	NR	NR	NR	NR
site7	NR	NR	NR	NR	NR
site8	NR	NR	NR	NR	NR
site9	NR	NR	NR	NR	NR
site10	NR	NR	NR	NR	NR
site11	NR	NR	NR	NR	NR
site12	NR	NR	NR	NR	NR
site13	NR	NR	NR	NR	NR
site14	NR	NR	NR	NR	NR
site15	NR	NR	NR	NR	NR
site16	NR	NR	NR	NR	NR
site17	NR	NR	NR	NR	NR

Patient 22

Pacing Site	Ischemia percentage 1.75%	1.26%	1.05%	0.64%	0.18%
site1	NR	NR	NR	NR	NR
site2	NR	NR	NR	NR	NR
site3	NR	NR	NR	NR	NR
site4	NR	NR	NR	NR	NR
site5	NR	NR	NR	NR	NR
site6	NR	NR	NR	NR	NR
site7	NR	NR	NR	NR	NR
site8	NR	NR	NR	NR	NR
site9	NR	NR	NR	NR	NR
site10	NR	NR	NR	NR	NR
site11	NR	NR	NR	NR	NR
site12	NR	NR	NR	NR	NR
site13	NR	NR	NR	NR	NR
site14	NR	NR	NR	NR	NR
site15	NR	NR	NR	NR	NR
site16	NR	NR	NR	NR	NR
site17	NR	NR	NR	NR	NR

Patient 23

Pacing Site	Ischemia percentage 27.64 %	22.04%	19.39%	12.38%	4.18%
site1	UR at s2	UR at s3	UR at s3	UR at s2	NR
site2	UR at s2	UR at s3	R at s4	NR	NR
site3	UR at s3	UR at s3	UR at s4	NR	NR
site4	R at s4	R at s4	R at s4	R at s4	NR
site5	R at s4	UR at s4	NR	NR	NR
site6	NR	NR	R at s4	NR	NR
site7	NR	NR	NR	NR	NR
site8	UR at s3	R at s4	R at s4	NR	NR
site9	R at s3	R at s3	R at s4	UR at s4	NR
site10	UR at s3	R at s4	R at s4	NR	NR
site11	NR	NR	NR	NR	NR
site12	R at s3	R at s4	R at s3	NR	NR
site13	NR	NR	R at s2	UR at s2	UR at s4
site14	UR at s3	R at s4	R at s4	NR	NR
site15	UR at s3	R at s4	NR	NR	NR
site16	R at s4	R at s4	NR	NR	NR
site17	UR at s4	NR	NR	NR	NR

Patient 24

**Pacing Site**	**Ischemia percentage** 9.81%	**7.56%**	**6.56%**	**4.11%**	**1.57%**
site1	NR	NR	NR	NR	NR
site2	NR	NR	NR	NR	NR
site3	UR at s3	NR	NR	NR	NR
site4	NR	NR	NR	NR	NR
site5	NR	NR	NR	NR	NR
site6	NR	NR	NR	NR	NR
site7	NR	NR	NR	NR	NR
site8	NR	NR	NR	NR	NR
site9	UR at s3	NR	NR	NR	NR
site10	NR	NR	NR	NR	NR
site11	NR	NR	NR	NR	NR
site12	NR	NR	NR	NR	NR
site13	NR	NR	NR	NR	NR
site14	NR	NR	NR	NR	NR
site15	NR	NR	NR	NR	NR
site16	UR at s4	NR	NR	NR	NR
site17	NR	NR	NR	NR	NR

Patient 25

**Pacing Site**	**Ischemia percentage** 6.03%	**4.59%**	**3.94%**	**2.48%**	**0.94%**
site1	NR	NR	NR	NR	NR
site2	NR	NR	NR	NR	NR
site3	NR	NR	NR	NR	NR
site4	NR	NR	NR	NR	NR
site5	NR	NR	NR	NR	NR
site6	NR	NR	NR	NR	NR
site7	NR	NR	NR	NR	NR
site8	NR	NR	NR	NR	NR
site9	NR	NR	NR	NR	NR
site10	NR	NR	NR	NR	NR
site11	NR	NR	NR	NR	NR
site12	NR	NR	NR	NR	NR
site13	NR	NR	NR	NR	NR
site14	NR	NR	NR	NR	NR
site15	NR	NR	NR	NR	NR
site16	NR	NR	NR	NR	NR
site17	NR	NR	NR	NR	NR

Patient 26

Pacing Site	Ischemia percentage 0 %
site1	NR
site2	NR
site3	NR
site4	NR
site5	NR
site6	NR
site7	NR
site8	NR
site9	NR
site10	NR
site11	NR
site12	NR
site13	NR
site14	NR
site15	NR
site16	NR
site17	NR

Patient 27

Pacing	Ischemia percentage				
Site	5.52%	4.03%	3.36%	1.94%	0.44%
site1	NR	NR	NR	NR	NR
site2	NR	NR	NR	NR	NR
site3	NR	NR	NR	NR	NR
site4	NR	NR	NR	NR	NR
site5	UR at s3	NR	NR	NR	NR
site6	NR	NR	NR	NR	NR
site7	NR	NR	NR	NR	NR
site8	NR	NR	NR	NR	NR
site9	NR	NR	NR	NR	NR
site10	NR	NR	NR	NR	NR
site11	NR	NR	NR	NR	NR
site12	NR	NR	NR	NR	NR
site13	NR	NR	NR	NR	NR
site14	NR	NR	NR	NR	NR
site15	NR	NR	NR	NR	NR
site16	NR	NR	NR	NR	NR
site17	NR	NR	NR	NR	NR

Patient 28

**Pacing Site**	**Ischemia percentage** 11.10%	**8.67%**	**7.5%**	**4.59%**	**1.43 %**
site1	R at s3	UR at s3	NR	NR	NR
site2	UR at s4	NR	NR	NR	NR
site3	NR	NR	NR	NR	NR
site4	NR	NR	NR	NR	NR
site5	NR	NR	NR	NR	NR
site6	NR	NR	NR	NR	NR
site7	UR at s4	NR	NR	NR	NR
site8	NR	NR	NR	NR	NR
site9	NR	NR	NR	NR	NR
site10	NR	NR	NR	NR	NR
site11	NR	NR	NR	NR	NR
site12	NR	NR	NR	NR	NR
site13	NR	NR	NR	NR	NR
site14	NR	NR	NR	NR	NR
site15	NR	NR	NR	NR	NR
site16	NR	NR	NR	NR	NR
site17	NR	NR	NR	NR	NR

Patient 29

**Pacing Site**	**Ischemia percentage** 9.67 %	**7.05%**	**5.98%**	**3.57%**	**1.03%**
site1	NR	NR	NR	NR	NR
site2	NR	NR	NR	NR	NR
site3	NR	NR	NR	NR	NR
site4	NR	NR	NR	NR	NR
site5	NR	NR	NR	NR	NR
site6	NR	NR	NR	NR	NR
site7	NR	NR	NR	NR	NR
site8	NR	NR	NR	NR	NR
site9	NR	NR	NR	NR	NR
site10	NR	NR	NR	NR	NR
site11	NR	NR	NR	NR	NR
site12	NR	NR	NR	NR	NR
site13	NR	NR	NR	NR	NR
site14	NR	NR	NR	NR	NR
site15	NR	NR	NR	NR	NR
site16	NR	NR	NR	NR	NR
site17	NR	NR	NR	NR	NR

Patient 30

Pacing Site	Ischemia percentage 16.35 %	12.98%	11.5%	7.75%	3.59 %
site1	UR at s3	NR	NR	NR	NR
site2	UR at s3	UR at s4	NR	NR	NR
site3	NR	NR	NR	NR	NR
site4	NR	NR	NR	NR	NR
site5	UR at s4	NR	NR	NR	NR
site6	NR	NR	NR	NR	NR
site7	NR	NR	NR	NR	NR
site8	NR	UR at s4	NR	NR	NR
site9	R at s2	NR	NR	NR	NR
site10	NR	UR at s4	UR at s4	NR	NR
site11	NR	NR	NR	NR	NR
site12	UR at s4	NR	NR	NR	NR
site13	NR	NR	NR	NR	NR
site14	NR	NR	NR	NR	NR
site15	UR at s4	NR	NR	NR	NR
site16	UR at s4	NR	NR	NR	NR
site17	NR	UR at s4	NR	NR	NR

Table S5: Supplementary Results: Feature statistics ~~and arrhythmia~~ in baseline and augmented populations grouped by simulation results (i.e. arrhythmia or no arrhythmia).

	Mean	Median	Standard Deviation
**segment specific myocardial volume, baseline (ml)**	6.162	5.716	2.342
no arrhythmia	5.946	5.442	2.314
arrhythmia	6.955	6.744	2.271
**segment specific myocardial volume, augmented (ml)**	6.183	5.965	2.193
no arrhythmia	6.045	5.774	2.108
arrhythmia	7.11	6.776	2.507
**segment specific ischemia percentage, baseline (%)**	9.097	0.0	19.465
no arrhythmia	6.008	5.442	2.314
arrhythmia	20.459	6.744	2.271
**segment specific ischemia percentage, augmented (%)**	6.771	0.0	15.472
no arrhythmia	4.747	5.774	2.108
arrhythmia	20.43	6.776	2.507
**global myocardial volume, baseline (ml)**	104.75	101.282	31.29
no arrhythmia	100.743	5.442	2.314
arrhythmia	119.494	6.744	2.271
**global myocardial volume, augmented (ml)**	105.105	104.617	28.018
no arrhythmia	103.007	5.774	2.108
arrhythmia	119.264	6.776	2.507
**global ischemic volume, baseline (ml)**	12.208	7.402	13.149
no arrhythmia	7.739	5.442	2.314
arrhythmia	28.648	6.744	2.271
**global ischemic volume, augmented (ml)**	9.004	5.25	10.268
no arrhythmia	6.681	5.774	2.108
arrhythmia	24.683	6.776	2.507

Table S6: Results. ML model precision ($\frac{TP}{TP+FP}$) at a threshold of 0.5 for baseline and augmented populations.

Model	Mean	Standard Deviation	Max	Min
k-nearest neighbors, baseline	0.66722	0.07945	0.85714	0.46667
k-nearest neighbors, augmented	0.61989	0.05890	0.76119	0.47692
support vector machine, baseline	0.78436	0.08839	0.95652	0.50000
support vector machine, augmented	0.66937	0.06878	0.89189	0.52083
logistic regression, baseline	0.78929	0.08428	0.95455	0.55000
logistic regression, augmented	0.67023	0.05644	0.83333	0.50000
decision tree, baseline	0.68617	0.08527	0.90476	0.47500
decision tree, augmented	0.60104	0.06998	0.81481	0.43103
xgboost, baseline	0.63467	0.08020	0.86957	0.41026
xgboost, augmented	0.57868	0.05438	0.70968	0.45238
3 hidden layer neural network, baseline	0.68706	0.11314	1.00000	0.42553
3 hidden layer neural network, augmented	0.66096	0.10036	1.00000	0.45455
4 hidden layer neural network, baseline	0.69400	0.11412	0.95652	0.45902
4 hidden layer neural network, augmented	0.66445	0.08659	0.88571	0.47761

Table S7: Results. ML model sensitivity ($\frac{TP}{TP+FN}$) at a threshold of 0.5 for baseline and augmented populations.

Model	Mean	Standard Deviation	Max	Min
k-nearest neighbors, baseline	0.58591	0.08545	0.76667	0.34286
k-nearest neighbors, augmented	0.48591	0.05361	0.62667	0.4
support vector machine, baseline	0.5067	0.07531	0.71429	0.32258
support vector machine, augmented	0.37214	0.04725	0.50667	0.23913
logistic regression, baseline	0.49999	0.0739	0.68571	0.32258
logistic regression, augmented	0.41027	0.04754	0.50617	0.31111
decision tree, baseline	0.54764	0.10627	0.85294	0.2963
decision tree, augmented	0.45468	0.12446	0.78049	0.14607
xgboost, baseline	0.56039	0.08228	0.71429	0.36111
xgboost, augmented	0.47688	0.05365	0.64	0.36471
3 hidden layer neural network, baseline	0.58673	0.1278	0.96296	0.09091
3 hidden layer neural network, augmented	0.42147	0.1432	0.76623	0.03488
4 hidden layer neural network, baseline	0.5934	0.14594	0.96552	0.11765
4 hidden layer neural network, augmented	0.42102	0.14209	0.75556	0.11702

Table S8: Supplementary Results: P-values for the t-test (second column) for increase in model accuracy when training on the augmented population compared to baseline, and f-test (third column) for decreased model variance when training on the augmented population compared to baseline.

Model	Mean Model Accuracy	Accuracy Variance
k-nearest neighbors	9.27e-36	1.11e-16
support vector machine	3.45e-26	3.88e-15
logistic regression	2.18e-29	8.88e-16
decision tree	2.34e-38	2.09e-12
xgboost	6.22e-41	1.11e-16
3 hidden layer neural network	4.26e-34	2.22e-16
4 hidden layer neural network	8.85e-31	1.11e-16

Supplementary Materials

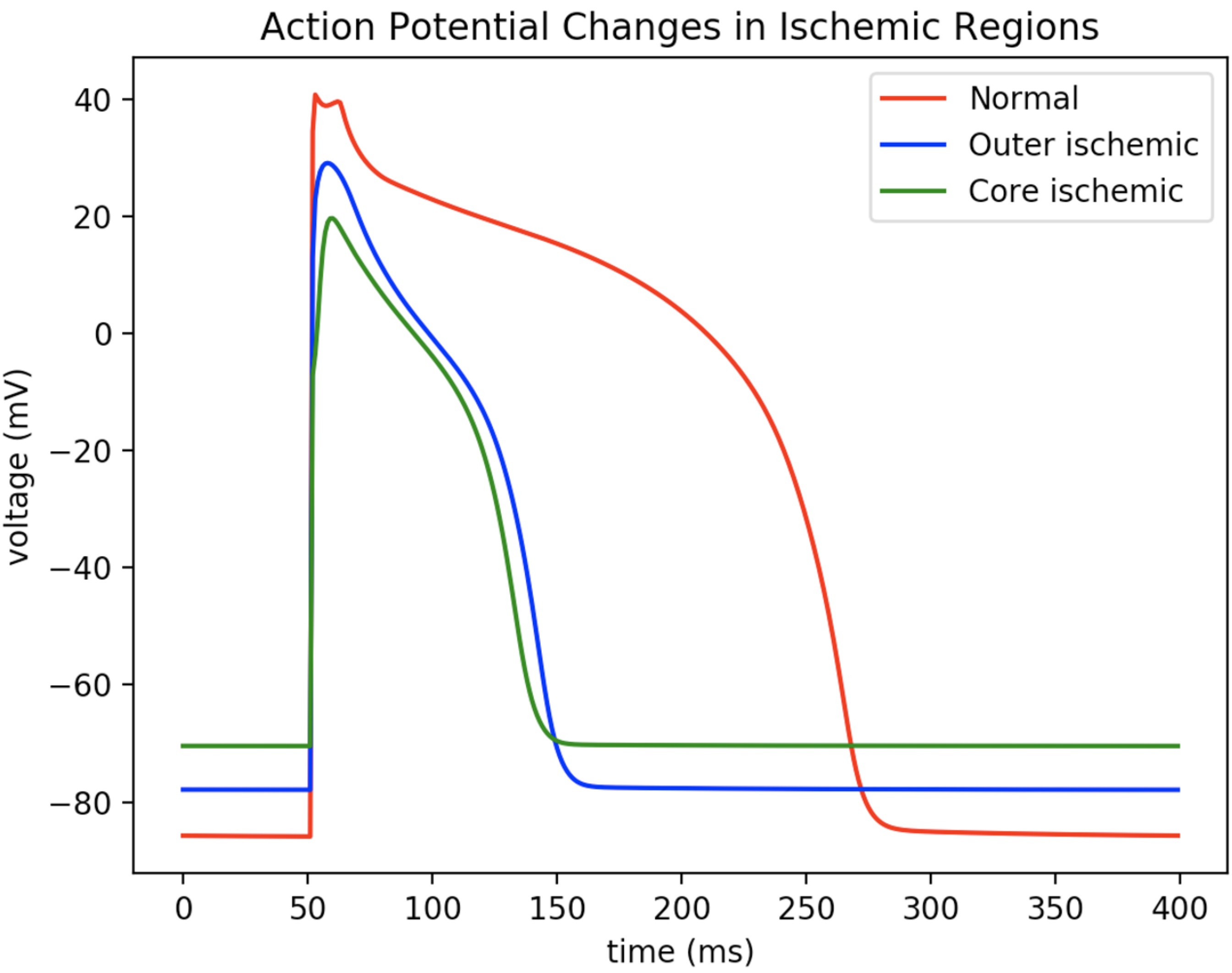

Figure S1: Supplementary Results: Action potential traces of healthy myocardium, outermost ischemic layer, and innermost ischemic layer.

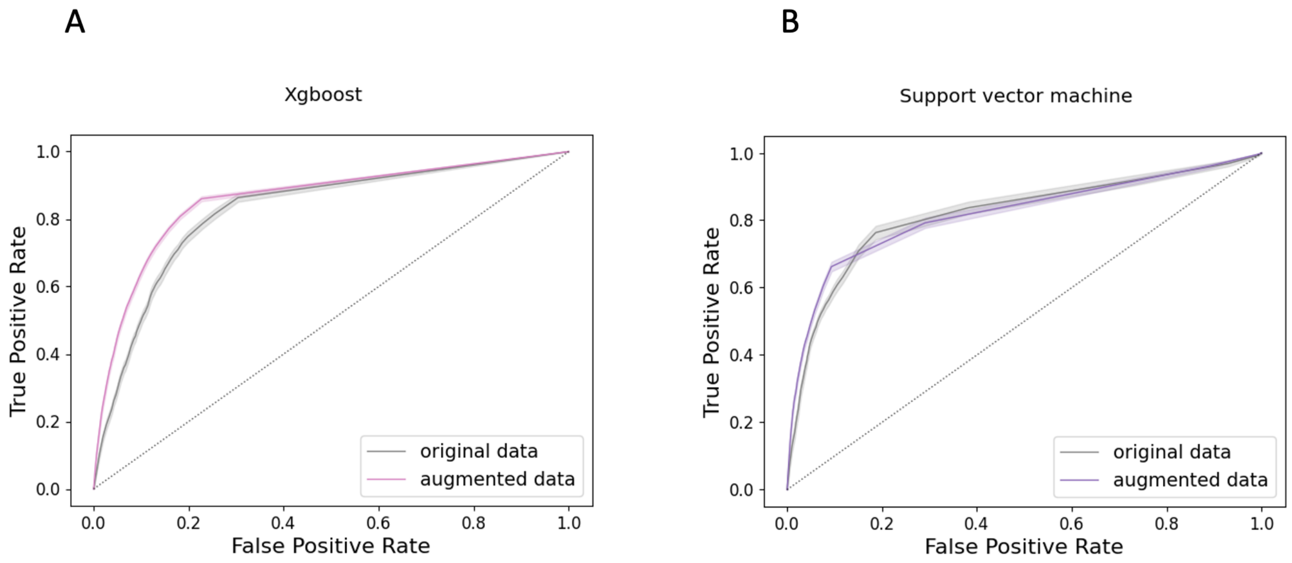

Figure S2: Supplementary Results: ROC curves with 95 % confidence interval for XGBoost and Support vector machine, comparing models trained on augmented and baseline population. True positive rate = $TP/(TP+FN)$, false positive rate = $FP/(FP+TN)$. ~~Machine learning model performance, additional models.~~

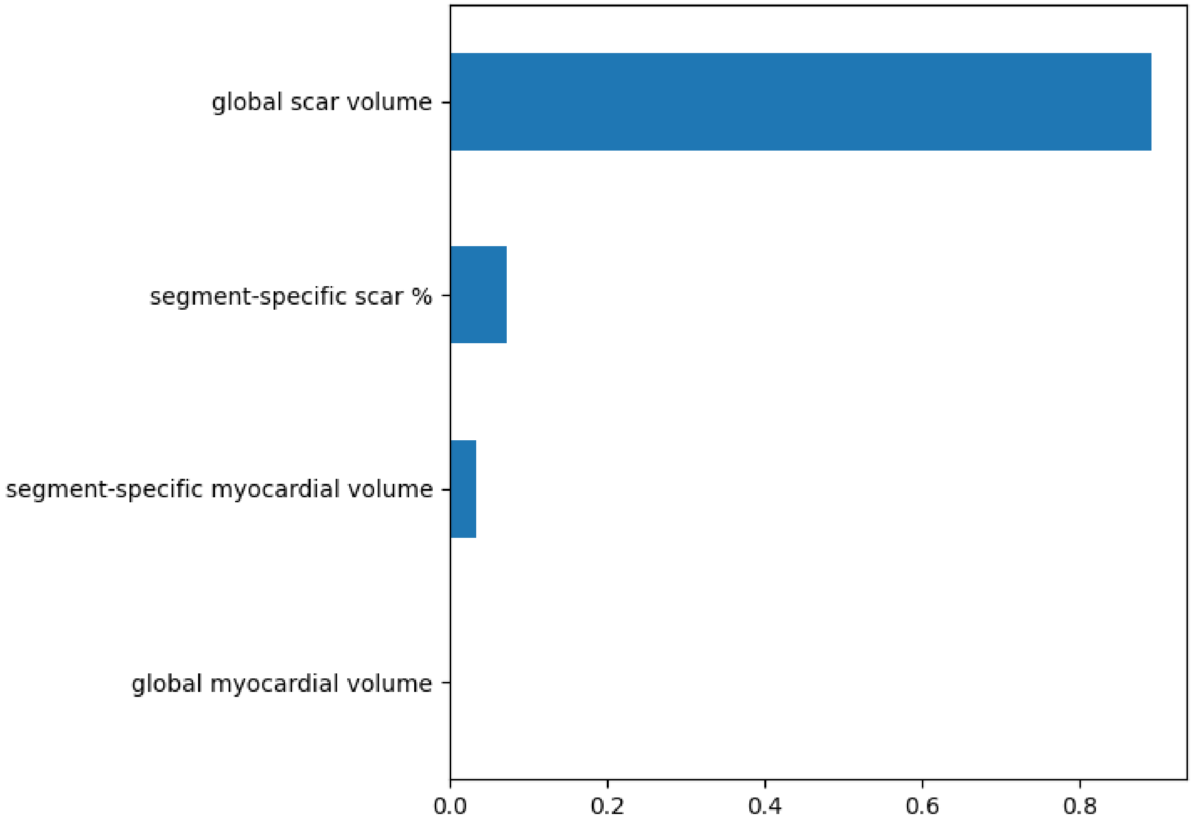

Figure S3: Supplementary Results: Average decision tree feature importance for trees trained on baseline population. The importance of a feature is calculated according to the gini importance, $Imp()$, which for a feature X_m, is given by adding up the weighted impurity decreases $p(t)\Delta i(s_t, t)$ for all nodes t where X_m is used, $Imp(X_m) = \Sigma_{t:v(s_t)=X_m} p(t)\Delta i(s_t, t)$. $p(t)$ is the proportion of samples reaching node t, $v(st)$ is the variable used in split s_t and $i(t)$ is the gini impurity measure,$i(t) = \Sigma_{j=1}^{J} \phi_j(t)(1 - \phi_j(t))$ where $\phi_j(t)$ denotes the class frequency for class label $j \in [1, J]$ in a node t.

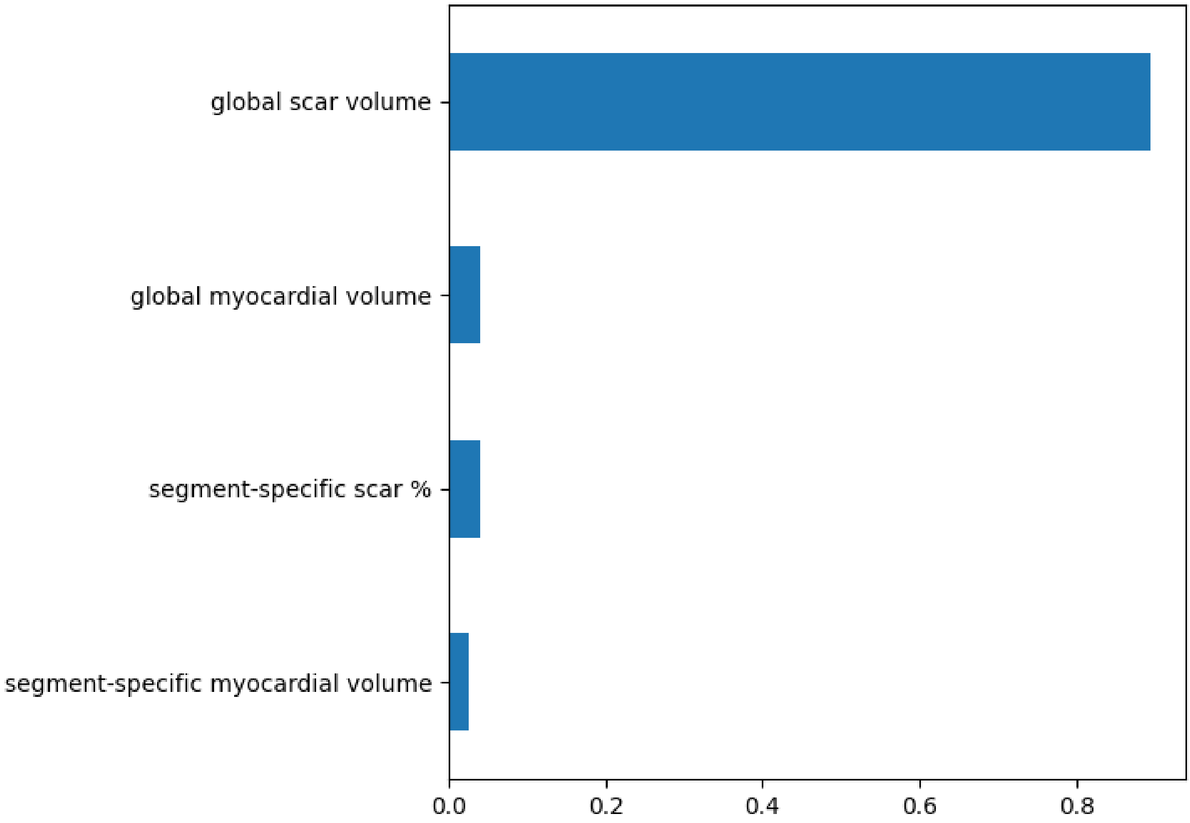

Figure S4: Supplementary Results: Average decision tree feature importance for trees trained on augmented population.The importance of a feature is calculated according to the gini importance, $Imp()$, which for a feature X_m, is given by adding up the weighted impurity decreases $p(t)\Delta i(s_t, t)$ for all nodes t where X_m is used, $Imp(X_m) = \Sigma_{t:v(s_t)=X_m} p(t)\Delta i(s_t, t)$. $p(t)$ is the proportion of samples reaching node t, $v(st)$ is the variable used in split s_t and $i(t)$ is the gini impurity measure,$i(t) = \Sigma_{j=1}^{J} \phi_j(t)(1 - \phi_j(t))$ where $\phi_j(t)$ denotes the class frequency for class label $j \in [1, J]$ in a node t.

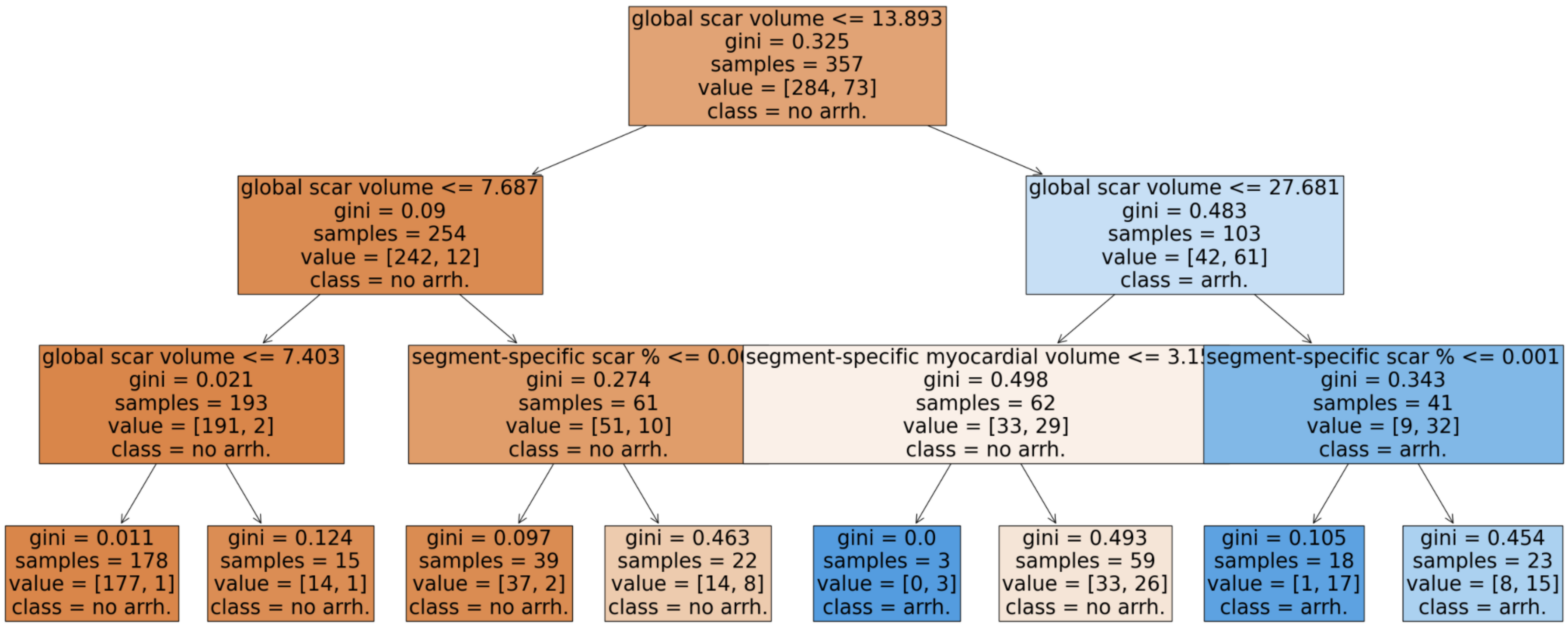

Figure S5: Supplementary Results: Decision tree - baseline population. Each box is a node. In each node, a split is made based on obtaining minimal gini impurity, defined by $i(t) = \Sigma_{j=1}^{J}\phi_j(t)(1-\phi_j(t))$. The first line in each node describes the criterion that the split is made on. *gini* is the value of the gini impurity with the current split. *samples* outputs how many samples are placed in the different classes (arrhythmia or no arrhythmia) based on the split, and thus how many samples are forwarded into different child nodes. *class* outputs which class the of the majority of samples are labeled as.

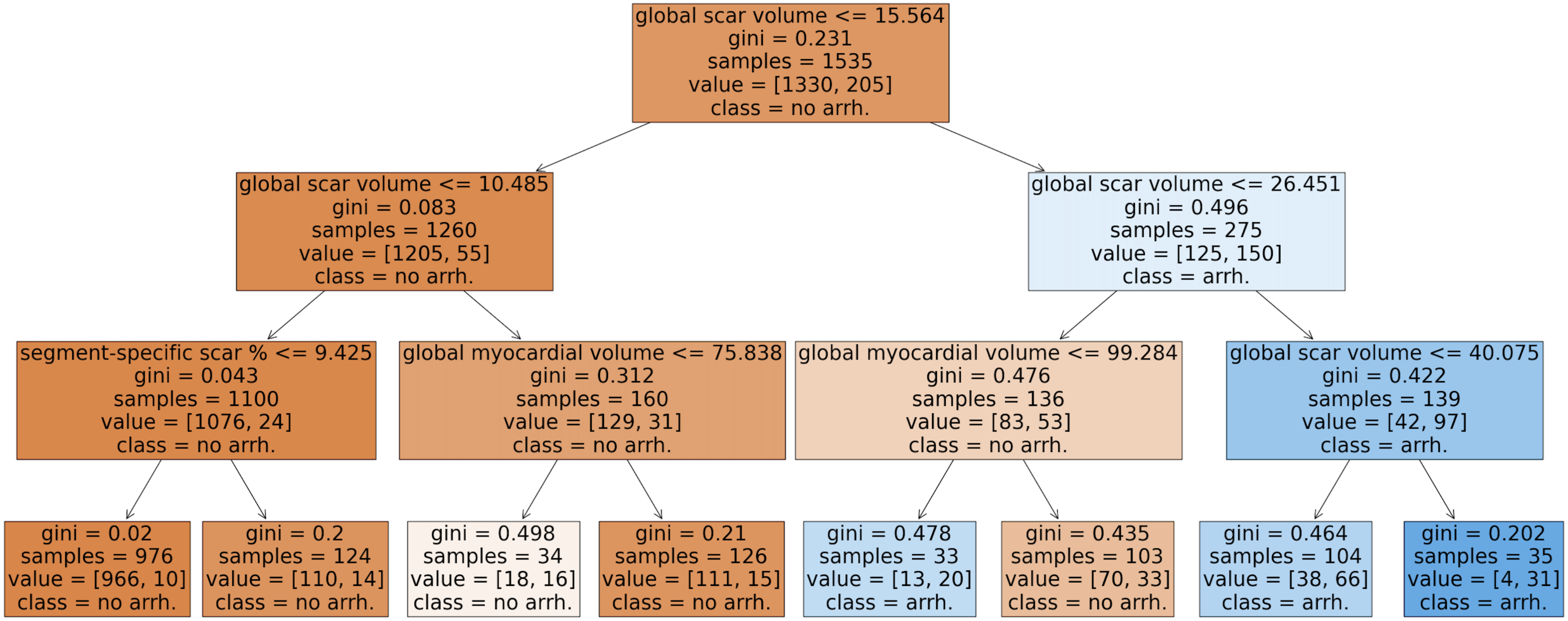

Figure S6: Supplementary Results: Decision tree - augmented population. Each box is a node. In each node, a split is made based on obtaining minimal gini impurity, defined by $i(t) = \Sigma_{j=1}^{J} \phi_j(t)(1 - \phi_j(t))$. The first line in each node describes the criterion that the split is made on. *gini* is the value of the gini impurity with the current split. *samples* outputs how many samples are placed in the different classes (arrhythmia or no arrhythmia) based on the split, and thus how many samples are forwarded into different child nodes. *class* outputs which class the of the majority of samples are labeled as.
